# A brain-constrained neural model of cognition and language with NEST: transitioning from the Felix framework

**DOI:** 10.1007/s11571-026-10415-5

**Published:** 2026-02-06

**Authors:** Maxime Carriere, Fynn Dobler, Hans Ekkehard Plesser, Agata Feledyn, Rosario Tomasello, Thomas Wennekers, Friedemann Pulvermüller

**Affiliations:** 1 Department of Philosophy and Humanities Brain Language Laboratory, WE4 Freie Universität Berlin, 14195 Berlin, Germany; 2 Cluster of Excellence’ Matters of Activity. Image Space Material’, Humboldt Universität zu Berlin, 10099 Berlin, Germany; 3 School of Mind and Brain, Humboldt Universität zu Berlin, 10117 Berlin, Germany; 4 Einstein Center for Neurosciences, 10117 Berlin, Germany; 5 School of Engineering, Computing and Mathematics, Faculty of Science and Engineering, University of Plymouth, PL4 8AA Plymouth, UK; 6 Department of Data Science, Faculty of Science and Technology, Norwegian University of Life Sciences, Ås, Norway; 7 Institute for Advanced Simulation (IAS-6), Research Centre Jülich, Jülich, Germany; 8 Käte Hamburger Kolleg: Cultures of Research (c:o/re), RWTH Aachen University, Aachen, Germany

**Keywords:** Brain-constrained model, Language model, Hebbian learning, NEST, Neural network

## Abstract

We introduce a brain-constrained neurocomputational model designed to simulate higher cognitive functions of the human brain, implemented using NEST, a widely used open-source simulator optimised for high-performance spiking neural network simulations. Previously implemented in the custom-built C-based Felix simulation library, transitioning the model to NEST enhances accessibility, reproducibility, and computational efficiency. At the cellular level, the model comprises spiking excitatory neurons and local inhibitory neurons, whereas at the network level, it replicates the structural and functional organisation of 12 cortical regions spanning frontal, temporal, and occipital cortices, along with their associated inter-area connectivity. Additionally, global inhibition mechanisms and neuronal noise are integrated. Learning in the model follows biologically plausible Hebbian plasticity principles, incorporating both long-term potentiation and long-term depression. To validate the NEST implementation, we replicated previous simulation findings obtained with the Felix-based model. The new implementation successfully reproduced the same topographical distribution of cell assemblies following associative learning of object and action words within action and perception systems, replicating a range of previous neuroimaging results. Although the NEST model produced larger cell assemblies than Felix, the overall topographical patterns remained similar, indicating preservation of fundamental network characteristics. Moreover, the transition to NEST significantly enhanced computational efficiency, reducing simulation runtime nearly sixfold compared to Felix. This improvement in computational speed is crucial for expanding the model to include additional cortical regions, such as extending to the right hemisphere, which necessitates increased computational resources.

**Supplementary Information:**

The online version contains supplementary material available at 10.1007/s11571-026-10415-5.

## Introduction

In recent decades, brain-constrained computational models have emerged as powerful tools for understanding the neural mechanisms underlying complex cognitive processes, such as language, memory, and perception (Bibbig et al. 1995; Eliasmith and Anderson 2003; Sommer and Wennekers 2003; Wennekers et al. 2006; Garagnani et al. 2007; Garagnani and Pulvermüller 2016; van Gerven and Bohte 2017; Kriegeskorte and Douglas 2018; Tomasello et al. 2018; Dobler et al. 2024, 2025; Nguyen et al. 2024; Carriere et al. 2024, 2025). These models integrate biological and plausible principles by mimicking key aspects of neural anatomy, connectivity, and dynamics, allowing researchers to explore how specific features of the brain’s structure shape its functional properties (van Albada et al. 2022; Pronold et al. 2024). By grounding theoretical hypotheses in biologically plausible frameworks, these models can provide a bridge between experimental data and computational neuroscience (Deco et al. 2008; Carandini 2012; Einevoll et al. 2019; Pulvermüller et al. 2021).

However, the rapid expansion of this field has exposed critical challenges, particularly regarding reproducibility, scalability, and accessibility (Poldrack et al. 2017; Marwick et al. 2018; Plesser 2018). Many existing models lack accessibility as their code is often unavailable or difficult to reproduce due to incomplete documentation of training procedures and parameters. This limits replication and extensions of modelling approaches, and their adoption by the broader research community (Goodman and Brette 2009; Ngai 2022). These barriers hinder progress and underscore the need for open, scalable tools to support the reproducibility and democratisation of brain-constrained modelling.

A recent variant of brain-constrained neural network models, the ‘Brain-constrained Perception Action Model of Cognition and Language’ or BPAM-o-CAL, has been extensively applied to implement higher human cognitive capacities, including the emergence of verbal working memory, building of symbolic representations, the formation and processing of concepts and different types of meaning (Garagnani and Pulvermüller 2016; Tomasello et al. 2017, 2018; 2025; Constant et al. 2023; Henningsen-Schomers and Pulvermüller 2022; Dobler et al. 2024). Specific studies covered a range of topics, including: (i) the interaction between attention processes and language (Garagnani et al. 2008); (ii) neural plasticity in congenitally blind populations (Tomasello et al. 2019, 2024); (iii) fast mapping of words to referents (Constant et al. 2023; Tomasello 2025); (iv) neuroanatomical constraints enabling memory formation for spoken words (Schomers et al. 2017; Carriere et al. 2024); (v) learning and representation building for concrete conceptual categories (Nguyen et al. 2024); (vi) language modulating color perception (Tomasello et al. under review); and (vii) concrete versus abstract concepts and symbolic meanings (Henningsen-Schomers and Pulvermüller 2022; Henningsen-Schomers et al. 2022; Dobler et al. 2024). Notably, all of the aforementioned studies were developed within the same research group using the Felix neural simulation framework (Wennekers et al. 2006; for recent review see Pulvermüller 2023).

Initially designed for small-scale network simulations and dynamical systems, Felix was later adapted to model six cortical areas known to be of particular relevance to language, which include the left auditory and articulatory areas and the so-called ‘language regions’ of Broca and Wernicke (Wennekers et al. 2006; Garagnani et al. 2008). This 6-area model was subsequently expanded to include 12 cortical areas, integrating visual, motor and adjacent prefrontal and anterior-temporal regions to provide a more comprehensive framework for studying semantic learning and symbolic processing (Garagnani and Pulvermüller 2016; Tomasello et al. 2017, 2018, 2019; Henningsen-Schomers and Pulvermüller 2022; Dobler et al. 2024). The model integrates neuroanatomical and physiological principles across micro- and macro-levels of the human cortex, supporting biologically grounded simulations (Pulvermüller et al. 2021; 2023). Although several biological features may require further refinement, such as more realistic neural dynamics, precise temporal structure, and a biologically plausible excitatory–inhibitory balance, the model in its present form has nonetheless successfully reproduced a range of neuroimaging findings and provided mechanistic accounts for multiple linguistic phenomena (see Pulvermüller 2023; for review).

While these models have yielded critical insights into the neural basis of language processing, their reliance on Felix – an older simulator restricted to 32-bit Linux, has limited their accessibility, reproducibility, and scalability. This has created barriers for widespread adoption and independent validation, underscoring the need for a transition to an open-source, widely available alternative.

To address these challenges, we chose to re-implement the BPAM-o-CAL model in NEST (NEural Simulation Tool), a parallel open-source simulator designed for spiking neural networks (Gewaltig and Diesmann 2007; Plesser et al. 2022). By transitioning our model to NEST, we not only take advantage of its technical strengths but also contribute to the model sharing and re-use in advanced brain-constrained modelling, addressing ongoing concerns about reproducibility and ensuring that biologically realistic simulations remain at the forefront of cognitive neuroscience (Nordlie et al. 2009; Rougier et al. 2017; Plesser 2018).

The primary objective of this study is to validate the re-implementation of the BPAM-o-CAL to NEST by replicating key results from a seminal neurocomputational study on semantic learning in a spiking network anatomically aligned with relevant cortical structures (Pandya 1995; Pulvermüller 1999; Fadiga et al. 2002; Dum and Strick 2005; Rilling 2014). This previous study investigated essential features of semantic circuits and word acquisition, demonstrating the emergence of distributed, category-specific representations across cortical areas for action and object words (Tomasello et al. 2018). Specifically, neural populations responsive to action words were predominantly found in the motor system, particularly in the lateral primary motor and pre-motor cortex, areas M1L and PML, whereas those for object words were more prevalent in the visual system, especially in the primary visual cortex, area V1, and temporo-occipital cortex, area TO. To investigate the formation of these semantic circuits, each network implementation was subject to a learning phase in which articulatory-auditory activity patterns were presented alongside activation patterns in the primary visual or the dorsal motor cortex. This simulated spoken word learning in the context of either object perception (for object words) or motor action execution (for action words) (Garagnani and Pulvermüller 2016; Tomasello et al. 2017, 2018, 2024; Constant et al. 2023; Carriere et al. 2024). Learning was governed by Hebbian plasticity, incorporating both long-term potentiation and depression, ensuring that connections strengthened or weakened based on correlated activity.

In the present, we provide a precise description of the neural model and systematically compare results obtained with Felix and NEST across multiple levels of analysis. This includes (i) single-neuron analyses, enabling direct validation of the spiking behaviour and adaptation properties of the model, and (ii) larger-scale network analyses. The latter is crucial for confirming that NEST implementation replicates the semantic category specificity observed in the Felix implementation – namely, the predominant emergence of action-word circuits in motor regions and object-word circuits in visual regions, as documented in a range of neuroimaging studies (Binder et al. 2011; Pulvermüller 2013). While the underlying models in both Felix and NEST are formulated using the same differential equations and implement the same neurocomputational principles, achieving a perfect 1:1 numerical match is not always feasible due to implementation-level differences. These include explicit enforcement of synaptic delays in NEST, which were absent in Felix. Such differences can result in shifts in spike timing and activation dynamics that accumulate across simulation time. To compensate for these effects and ensure comparable network behaviour, several model parameters, particularly those governing inter-area connectivity and time constants, had to be adjusted. Replicating previous results would confirm that our Felix-based findings are robust across simulation environments and would establish a solid basis for future NEST simulations, enabling the incorporation of more detailed neural mechanisms and supporting multi-level modelling of cognitive functions.

## Materials and methods

The brain-constrained neural network model was initially developed using Felix, a C-based simulation tool designed for building neural networks and dynamical systems with a graphical user interface (GUI) that allows intuitive control of model parameters while running simulations (Wennekers et al. 2006). To validate the consistency of neuronal dynamics across implementations, we first constructed a single-neuron model in both Felix and NEST. This comparison focused on membrane potential dynamics, adaptation mechanisms, and spiking behaviour, ensuring alignment between the two frameworks. Building upon this validation, we implemented a multi-area cortical network in NEST, replicating the architecture and dynamics of the Felix-based model while leveraging Python’s flexibility and NEST’s computational efficiency. The 12-area network incorporated spiking excitatory and graded inhibitory neurons, activity-dependent synaptic plasticity, and structured long-range connectivity, capturing essential features of higher cognitive processing. These core components, established as fundamental for modelling cognitive functions (Pulvermüller et al. 2021), were systematically transferred to NEST, ensuring fidelity to the original Felix model:

- **Cortical areas** The model includes 12 cortical regions spanning auditory, visual, motor, and multimodal systems, organised into four functional subsystems:
  - **Auditory system** Primary auditory cortex (A1), auditory belt (AB), and auditory parabelt (PB).
  - **Visual system** Primary visual cortex (V1), temporo-occipital cortex (TO), and anterior-temporal lobe (AT).
  - **Articulatory system** Inferior motor (M1i), inferior premotor (PMi), and inferior prefrontal motor (PFi) cortices.
  - **Dorsolateral motor system** Dorsolateral motor (M1L), dorsolateral premotor (PML), and dorsolateral prefrontal (PFL) cortices.
- **Neuron model** The model employs excitatory spiking neurons to emulate the dynamics of cortical pyramidal cells, incorporating spatial and temporal summation of inputs, threshold-based “all-or-nothing” spiking, and neuronal adaptation. This approach captures key features of real neuronal activity, ensuring the model’s biological plausibility (Connors et al. 1982; Matthews 2001).
- **Regulation and control** Neural activity in the model is regulated by two types of inhibitory feedback mechanisms: local lateral inhibition within cortical areas and area-specific global inhibition. These mechanisms prevent runaway excitation and maintain physiological activity levels (Braitenberg 1978; Knoblauch and Palm 2002).
- **Local connectivity** Connectivity within each cortical region is sparse, random, and initially weak, with a bias toward short-range links. It includes excitatory neurons with both local and long-distance links and inhibitory neurons with local connections only, with interneurons having an even more restricted local reach than excitatory cells. This structure reflects the localised organisation of cortical circuits (Kaas 1997; Braitenberg and Schüz 2013).
- **Between-area global connectivity** Long-range cortico-cortical connections between regions are bidirectional, following neuroanatomical principles derived from experimental data. This connectivity enables the integration of sensory, motor, and multimodal information across cortical systems (Garagnani and Pulvermüller 2016).
- **Learning mechanisms** Synaptic plasticity is governed by a Hebbian learning rule, encompassing long-term potentiation (LTP) and long-term depression (LTD). These mechanisms simulate experience-dependent synaptic changes, allowing the formation of distributed neural assemblies representing learned associations (Artola and Singer 1993).
- **Neural noise** To mimic spontaneous neural activity and environmental variability, the model incorporates uncorrelated white noise at both the neuronal and input levels. This approach captures baseline firing rates and mimics activity from unmodelled brain areas and perceptual inputs (Deco et al. 2009).

Below, we provide a detailed description of the biological principles underlying the model, along with the corresponding mathematical formulations implemented in NEST, following the previous implementation in Felix (Tomasello et al. 2017, 2018; Constant et al. 2023; Dobler et al. 2024; Carriere et al. 2024). These principles were integrated into the brain-constrained neural network to simulate key mechanisms of the human cortex. We begin by outlining the macro-level implementation, covering the neuron population and cortical areas. This is followed by a description of the neuron model, including excitatory and inhibitory cell populations and their connectivity patterns, as well as the Hebbian synaptic plasticity rule, global inhibition, and additional neurophysiological constraints essential for simulating higher cognitive functions.

## Macro-level implementations

### Cortical areas

**Fig. 1:**
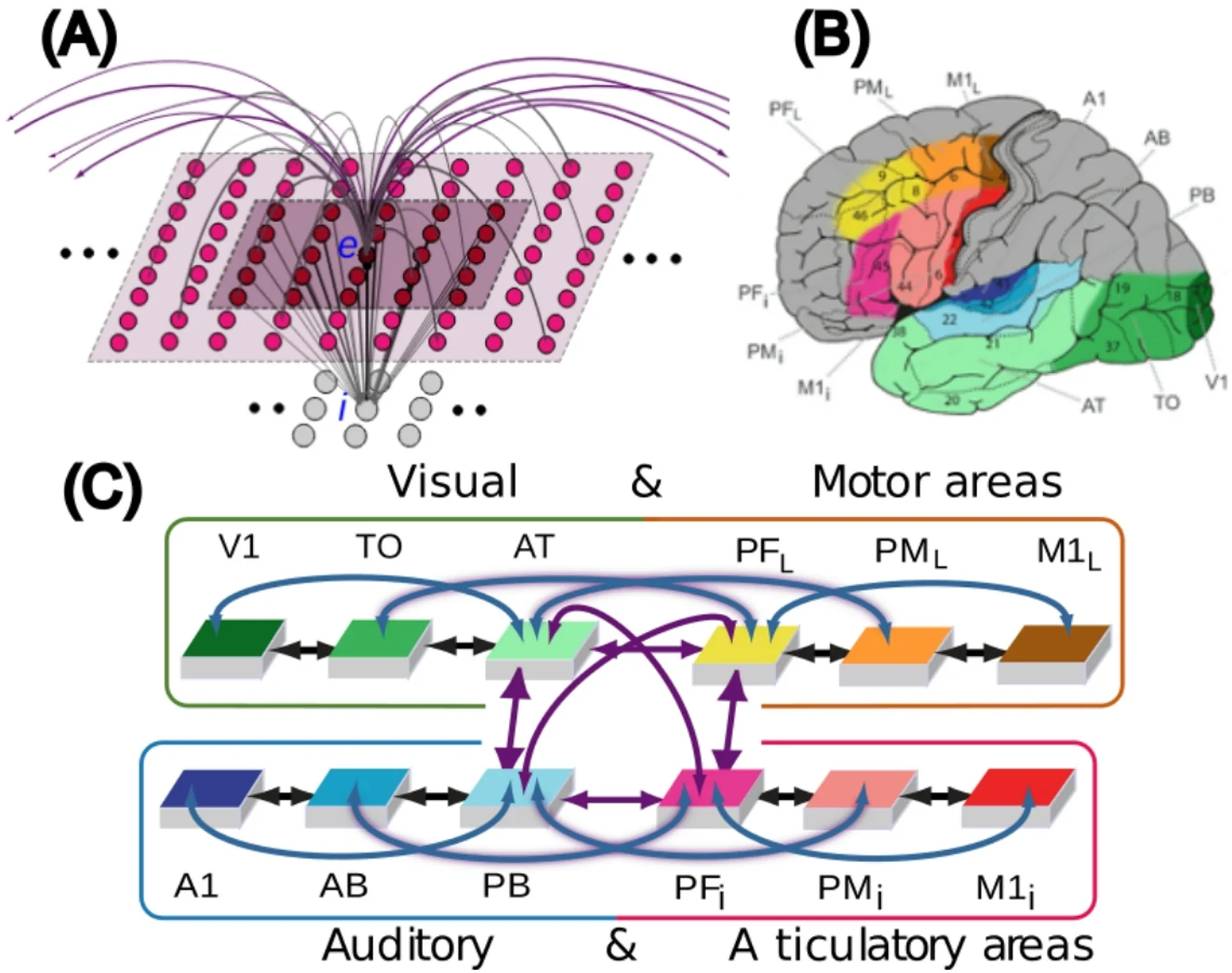
Structure and connectivity of the Brain-constrained Perception Action Model of Cognition and Language’ or BPAM-o-CAL. **A** Neuron-level connectivity of a single excitatory neuron (“e”) from one of the 7500 modeled neurons. Excitatory connections (gray) to and from “e” are random, sparse, and restricted to a local 19 × 19 neighborhood (light pink shaded area). Lateral inhibition is implemented through a dedicated inhibitory neuron (“i”), which inhibits “e” in proportion to the total excitatory input received from a 5 × 5 neighborhood (darker pink shaded area). Reciprocal inhibitory connections ensure local competition between excitatory neurons. **B** Cortical structure and connectivity of the left hemisphere as modeled in the network. Perisylvian regions include superior temporal auditory areas (blue) and inferior-frontal articulatory areas (red), while extrasylvian regions comprise dorsal motor areas (yellow/brown) and ventral visual areas (green). **C** Schematic representation of the network's inter-area connectivity. Adjacent areas within each system are connected via next-neighbour links (black arrows), while jumping links connect non-adjacent areas within the same system (blue arrows), or between two distinct systems (blue arrows with purple halo). Additionally, long-distance links (purple arrows) interconnect multimodal hub areas (PB, PFi, AT, and PFL), facilitating cross-system integration. This connectivity structure is based on neuroanatomical findings and previous computational models (adapted from Tomasello et al., 2018).

The model simulated 12 cortical regions critical for language processing, sensory integration, and motor control, organised into four functional systems: auditory, articulatory, visual and motor (See Fig. 1B–C). The auditory system included the primary auditory cortex (A1), auditory belt (AB), and parabelt (PB), which are essential for auditory information processing and phonological representation (Pulvermüller 1999; Pulvermüller and Fadiga 2010). The articulatory system comprised the inferior motor cortex (M1i), inferior premotor cortex (PMi), and multimodal prefrontal motor cortex (PFi), which are involved in motor control of speech articulatory movements (Zatorre et al. 1996; Fadiga et al. 2002).

Beyond these perisylvian language-related regions, the model incorporated six additional areas extending into visual and motor systems. The visual system included the primary visual cortex (V1), temporo-occipital cortex (TO), and anterior-temporal cortex (AT), supporting visual perception and integration (Ungerleider and Haxby 1994). The dorsolateral motor system consisted of the dorsolateral primary motor cortex (M1L), premotor cortex (PML), and prefrontal cortex (PFL), which are involved in arm and leg motor control (Deiber et al. 1991; Dum and Strick 2005). Each system contained one multimodal cortical region – PB, PFi, AT, and PFL, which served as connector hub areas that integrate sensory, motor, and multimodal information. These regions are considered “convergence zones” (Damasio 1989) or “connector hubs” (Heuvel and Sporns 2013), characterised by their dense connectivity to other cortical regions.

In both the original Felix implementation and the updated NEST framework, each cortical area consists of a 25 × 25 grid of excitatory spiking neurons (625 neurons), a corresponding 25 × 25 grid of inhibitory neurons modelled using rate-based dynamics, and a single area-specific global inhibitory unit (See Fig. 1A and Table 1). Although this 1:1 ratio between excitatory and inhibitory neurons deviates from the typical composition of the mammalian cortex (~ 80% excitatory, ~ 20% inhibitory; Azevedo et al. 2009; Herculano-Houzel 2009), it follows the design of previous simulations employing brain-constrained models (e.g., Garagnani et al. 2008; Garagnani and Pulvermüller 2016; Tomasello et al. 2018). This modelling choice was originally introduced for numerical convenience and because neurons within small local cortical patches (approximately micro-columnar scale) tend to fire in a highly similar way (Hubel 1995; Mountcastle 1997; Defelipe et al. 2012). Each modelled excitatory–inhibitory pair is therefore placed at this spatial scale: the spiking excitatory neuron is a typical exemplar of nearby excitatory cells, whose activity can be taken as indicative of the response of that patch, while the graded inhibitory neuron summarizes the collective influence of local inhibitory interneurons. In this sense, the network constitutes a spatially downsampled representation of cortex, in which many biological neurons in each patch are represented by a single excitatory–inhibitory pair in the model. The current NEST implementation retains this configuration for comparability with previous studies, but it also provides the flexibility to explore biologically more realistic ratios in future work.

**Table 1:** Number and types of neurons in one area

	One area		Neuron type	
	Felix	NEST	Felix	NEST
Excitatory Neurons	625	625	Spiking	Spiking
Inhibitory Neurons	625	625	Mean field	Mean field
Area Specific Inhibitory Neurons	1	1	Mean field	Mean field

## Micro-level implementation

### Neuron model

The model incorporates two types of neurons: excitatory spiking neurons and mean-field inhibitory neurons, each serving distinct functional roles within the network. Excitatory neurons are modelled as spiking leaky integrate-and-fire units, generating binary output (0 or 1) based on threshold activation, while inhibitory neurons operate under a mean-field approximation, producing graded responses that captures the collective influence of inhibitory interneurons within a cortical column. This distinction supports stable excitatory-inhibitory dynamics and controlled assembly growth, with excitatory neurons driving activity propagation and Hebbian association, and inhibitory neurons locally regulating network activity.

### Excitatory neurons

Excitatory neurons in the model approximate cortical pyramidal cell dynamics, including spatial and temporal summation of inputs, threshold-based “all-or-nothing” spiking, and neuronal adaptation. These neurons are modelled as integrate-and-fire units, with their activity governed by changes in membrane potential over time (Connors et al. 1982; Matthews 2001). The membrane potential *V* of an excitatory neuron *e* at time *t* is governed by the following first-order differential equation:

1 $$\:\tau\:\cdot\:\frac{dV(e,t)}{dt}=-V(e,t)+{k}_{1}\left(I\right(e,t)+{k}_{2}\eta\:(e,t\left)\right)$$

where:

- τ: Membrane time constant.
- I(e, t): Net synaptic input or current to the neuron (excitatory and inhibitory postsynaptic potentials, or EPSPs/IPSPs).
- η(e, t): Independent and identically distributed white noise processes with a uniform distribution over [− 0.5,0.5] representing baseline neural activity.
- k_1_, k_2_: Scaling constants.

The current I is calculated as:

2 $$\:I(\mathrm{x},\mathrm{t})=-{k}_{G}{G}_{A}\left(t\right)+\sum\:_{\mathrm{y}}\:{\mathrm{w}}_{\mathrm{x},\mathrm{y}}\cdot\:{\varphi\:}_{y}\left(t\right)$$

where:

• w(x, y): Synaptic weight from presynaptic neuron y to x.
• ϕ_y_(t): Output of presynaptic neuron y at time t (binary: 1 if firing, 0 otherwise).
• G_A_(t): Global inhibitory feedback specific to the cortical area A where the neuron x is located.
• k_G_: Scaling constant for global inhibition.
• The sum in Eq. 2 runs over all presynaptic neurons y connected to neuron x.

The output ϕ_y_(t) of a spiking excitatory neuron is binary and determined by the following threshold function:

3 $$\:{\varphi\:}_{y}\left(t\right)=\:\left\{\begin{array}{c}\:1\hspace{0.25em}\hspace{0.25em}\hspace{0.25em}\hspace{0.25em}\text{}\mathrm{i}\mathrm{f}\text{}\left(V\right(e,t)-\psi\:\alpha\:\omega\:(e,t\left)\right)>\text{}\mathrm{t}\mathrm{h}\mathrm{r}\mathrm{e}\mathrm{s}\mathrm{h}\text{}\\\:\:0\hspace{0.25em}\hspace{0.25em}\hspace{0.25em}\hspace{0.25em}\text{}\mathrm{o}\mathrm{t}\mathrm{h}\mathrm{e}\mathrm{r}\mathrm{w}\mathrm{i}\mathrm{s}\mathrm{e}\text{}\end{array}\right.$$

where:

- thresh: Firing threshold of the neuron.
- ω(e, t): Adaptation level of the neuron.
- ⍺: Scaling constant for adaptation level.
- *ψ*: Constant.

The scaling factor $\:\psi\:$ = 0.872 adjusts the contribution of adaptation in the spiking model to match firing behaviour observed in earlier mean-field implementations (Garagnani and Pulvermüller 2016; Tomasello et al. 2017). It was introduced when the Felix model was first extended to spiking dynamics (Garagnani et al. 2017; Tomasello et al. 2018), to ensure consistent threshold behaviour by compensating for differences in how adaptation influences excitability across excitatory neuron model types.

Neuronal adaptation is modelled by monitoring the cell’s recent firing activity:

4 $$\:\tau \:_{{ADAPT}} \:\cdot\:\frac{{d\omega \:(e,t)}}{{dt}}\: = - \omega \:(e,t) + \phi \:(e,t)$$

where:

- τ_ADAPT_ : Time constant of adaptation.
- ω(e, t): Low-pass filtered representation of recent firing activity.

A neuron’s average firing activity is used to compute the network’s Hebbian synaptic updates. The estimated instantaneous mean firing rate ω_E_(e, t) of an excitatory neuron is defined by:

5 $$\:{{\uptau\:}}_{\mathrm{F}\mathrm{a}\mathrm{v}\mathrm{g}}\:\cdot\:\frac{\mathrm{d}\:{{\upomega\:}}_{\mathrm{E}}\left(\mathrm{e},\:\mathrm{t}\right)\:\:}{\mathrm{d}\:\mathrm{t}}=-{{\upomega\:}}_{\mathrm{E}}\left(\mathrm{e},\:\mathrm{t}\right)\:+{\upvarphi\:}\left(\mathrm{e},\:\mathrm{t}\right)$$

where:

- τ_Favg_ : Time constant for firing rate estimation.
- ω_E(e, t)_ : Instantaneous mean firing rate of neuron e.
- ϕ(e, t) : Binary firing output.

This firing rate estimation provides a real-time measure of a neuron’s recent spiking activity, which is critical for implementing Hebbian learning rules in the model (See Learning Mechanisms Section). Unlike the classical integrate-and-fire neuron model, which explicitly tracks membrane potential and spike events, this approach focuses on estimating the mean firing rate over time, offering a simplified representation of the neuron’s spiking behaviour.

In the Felix implementation, spikes are modelled in discrete time steps, with the neuron’s firing activity being updated at each time step. The spike output ϕ(e, t) is treated as a binary value, where a neuron either fires (1) or does not fire (0) at a given time step. This discrete-time handling of spikes allows for efficient computation and aligns with the model’s temporal resolution, which operates in a stepwise fashion rather than simulating continuous spike events.

### Inhibitory neurons

Inhibitory neurons in the model follow a mean-field approximation to represent the collective activity of interneurons within a cortical column. Unlike excitatory neurons, which are modelled as spiking integrate-and-fire units with binary outputs (per simulated time-step), inhibitory neurons produce graded responses that reflect their overall activity level. This approach simplifies computations while preserving the essential inhibitory dynamics required for maintaining network stability. The membrane potential *V(i*,* t)* of an inhibitory neuron i at time t follows the same first-order differential equation as excitatory neurons described in Eq. (1). However, unlike excitatory neurons, inhibitory neurons do not include an adaptation mechanism, meaning ω(i, t) does not modulate their threshold or firing rate. The noise component η(i, t) is also absent for simplicity, i.e., k_2_ = 0, ensuring that spontaneous activity is solely driven by excitatory input. The net synaptic input or current *I(i*,* t)* is computed using Eq. (2) where inhibitory neurons receive excitatory drive from nearby pyramidal neurons and contribute to the homeostatic balance of excitation and inhibition within the cortical area. This local feedback inhibition supports competition among excitatory units by suppressing activity at each location, while the lateral excitatory connectivity mediates competition between neighbouring locations across the grid. By dynamically balancing excitation, local inhibition prevents excessive activity and helps maintain sparse and efficient neural representations, which are essential for cortical processing. In addition to local inhibitory circuits, the model also implements an area-specific global inhibitory mechanism, which ensures that the total firing activity of excitatory neurons within a cortical area remains within physiological levels (Braitenberg and Schüz 1998). This global inhibitory feedback is provided by a single graded-response inhibitory unit per area, which continuously monitors the total excitatory activity and proportionally inhibits all excitatory neurons within that area. The global inhibition signal G_A_(t) for a given area *A* at time *t* is defined by Eq. (6):

6 $$\:{{\uptau\:}}_{\mathrm{GLOB}}\cdot\:\frac{d{G}_{A}\left(t\right)}{dt}=-{G}_{A}\left(t\right)+{\sum\:}_{e\in\:A}{\upvarphi\:}\left(e,t\right)$$

where:

- τ_GLOB_ is the time constant for global inhibition, making it slower than local excitation-inhibition interactions.
- G_A_(t) represents the global inhibitory feedback signal for area A.
- The summation term computes the total firing rate of all excitatory neurons in the area.

This mechanism ensures that inhibition is applied proportionally across all excitatory neurons, maintaining balanced excitatory–inhibitory dynamics and preventing runaway excitation. It also provides a winner-takes-all dynamics – an area-wide competition that stabilises retrieved attractor patterns, which would be difficult to achieve with local inhibition alone. It follows the same formulation used in the original Felix-based models (Wennekers et al. 2006; Garagnani et al. 2008). Despite its abstraction, the global inhibition implementation is intended to mimic a simplified representation of the well-known distributed corticothalamic and reticular inhibitory systems that provide global gain control and attentional modulation across cortical territories (Jones, 2001; Sherman & Guillery, 1996). However, more work is needed to flesh out this interpretation (Table 2).

**Table 2:** Parameters for excitatory, inhibitory and global inhibitory neurons equations

	Excitatory Neurons		Inhibitory Neurons		Global inhibitory neurons	
	Felix	NEST	Felix	NEST	Felix	NEST
k_1_ (constant)	0.01	0.01	1	1	1	1
k_2_ (noise)	0.005	0.005	0	0	0	0
τ (Membrane time constant)	2.5	5	5	10	12	24
τ_ADAPT_ (Time constant of adaptation)	10	20	n/a	n/a	n/a	n/a
τ_FAVG_ (Time constant for firing rate estimation)	30	30	n/a	n/a	n/a	n/a
τ_GLOB_	n/a	n/a	n/a	n/a	12	24
*thresh (*Firing threshold)	0.18	0.18	n/a	n/a	n/a	n/a

## Connectivity

### Local connections

#### Excitatory to excitatory neurons

In the implementation, excitatory-to-excitatory (E-E) connectivity is generated as *pairwise Bernoulli trials* with a distance-dependent connection probability. Specifically, the probability *p(x*,* y)* that an excitatory neuron at location y connects to another excitatory neuron at location x follows a bivariate Gaussian spatial profile, meaning that nearby neurons are more likely to connect than distant ones. This approach corresponds to the distance-dependent connectivity schemes described in Senk et al. (2022). The probability function is given by the following formula (7):

7 $$p\left( {x,y} \right) = k_{C} e^{{\frac{{ - \frac{{\left( {x - \overline{x} } \right)^{2} }}{{\sigma _{x}^{2} }} + \frac{{\left( {y - \overline{y} } \right)^{2} }}{{\sigma _{y}^{2} }} + \frac{{2q\left( {x - \overline{x} } \right)\left( {y - \overline{y} } \right)}}{{\sigma _{x} \sigma _{y} }}}}{{2\left( {1 - q^{2} } \right)}}}}$$

where:

- *x* and *y*: Coordinates of the target neuron relative to the source neuron.
- *x̄* and ȳ: Mean coordinates in the x and y dimensions, representing the centre of the distribution.
- σ_x_ and σ_y_: Standard deviations along the *x* and *y* dimensions, which control the spread of the distribution in two orthogonal directions.
- *q*: Coefficient which accounts for the orientation of an un-isotropic Gaussian in the x-y-plane.
- *k*_*C*_: scaling factor.

Although the Eq. (7) appears more complex due to the inclusion of the parameters *x̄*, *ȳ* and q, we set *x̄* = *ȳ* = 0 and q = 0 in the implementation to yield an isotropic Gaussian distribution for all connection probabilities. This ensures that the connectivity pattern is symmetric around each neuron, without any directional bias. By doing so, the equation simplifies to a standard isotropic Gaussian. The more general form of the equation, with non-zero values for *x̄*, *ȳ*and q, is retained for flexibility. This allows the model to explore non-isotropic (anisotropic) Gaussian distributions, which may be relevant for specific types of connections, such as patch connections or other forms of spatially structured connectivity (Muir and Douglas 2011; Koestinger et al. 2017). These possibilities can be useful for investigating different network architectures that go beyond the isotropy assumption, enabling a more detailed analysis of spatial patterns in cortical circuits.

**Fig. 2:**
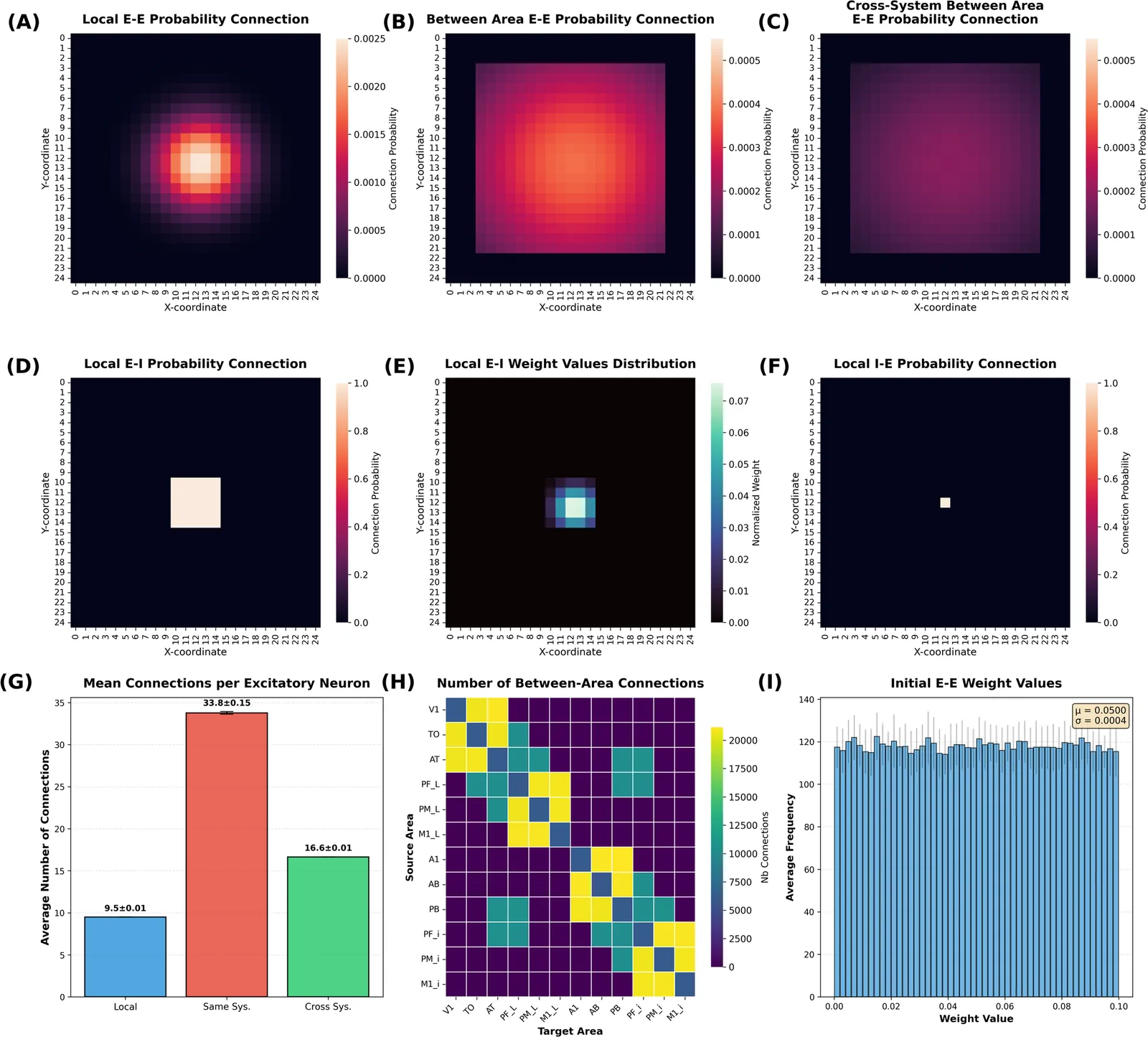
**A**–**C** Probability distributions of E-E connections for local (within-area) neurons, between-area neurons within the same functional system, and between-area neurons across distinct systems. The probability is shown for a neuron located at the center, with connections limited to a 19 × 19 grid around it. **D** Probability distributions of E-to-I connections. **E** Weight distributions of E-to-I connections. **F** Probability distributions of I-to-E connections **G** Average number of E-E connections per neuron for local connections (within-area, blue), between-area connections within the same functional system (orange), and cross-system connections (green). **H** Connectivity matrix illustrating area-to-area connections. Areas are connected to themselves, to other areas within the same functional system, and to secondary and central areas through cross-system connections. **I** Initial E-E synaptic weight distribution. The initial weights are uniformly distributed for local, between-area within the same system, and cross-system connections, with a maximum value of 0.1

This probability function is used to decide if a connection is made, where each pair of neurons is treated as an independent Bernoulli trial. Specifically, for each potential connection, a random value is sampled, and if it falls below *p(x*,* y)*, the connection is established. This probabilistic approach ensures realistic sparsity in the network’s connectivity structure. In our implementation, we set σ_x_ and σ_y_ = 3.2, which represents a relatively low spread of connectivity (See Fig. 2A). The k_C_ = 0.15 parameter, which scales overall connection probability, was adjusted to match the average number of excitatory connections per neuron in the Felix implementation. As a result, each excitatory neuron forms 9.5 connections with other excitatory neurons within the same area, with a standard error of 0.008. This configuration produces tightly clustered local connectivity, consistent with cortical architecture (Song et al. 2005; Perin et al. 2011). Each excitatory neuron can connect to other excitatory neurons within a 19 × 19 neighbourhood of the grid, which spans a radius of 9 cells around the neuron (See Fig. 2G) following previous studies (Bibbig et al. 1995; Wennekers et al. 2006).

The grid is treated as toroidal, meaning that neurons near the edges of the *25 × 25* grid can connect to neurons on the opposite edge. This approach ensures seamless connectivity without boundary effects. If a connection is formed, the synaptic weight is initialised as a random value drawn from a uniform distribution, with values bounded between 0 and a predefined upper limit w_u p_​= 0.1 (See Fig. 2I). The delay between excitatory and excitatory neurons is 1 time step, corresponding to twice the model’s resolution, which operates at 0.5 per time step.

#### Excitatory to inhibitory neurons

Each excitatory neuron connects to a 5 × 5 neighbourhood of inhibitory neurons with a probability of 1, ensuring that all inhibitory neurons within this region are connected to the source excitatory neuron (See Fig. 2, D). The bivariate Gaussian distribution (Eq. 7) is used to determine the synaptic weight values of E-to-I connections, meaning that neurons closer in space receive stronger weights, and more distant neurons receive weaker weights. To ensure tightly localised connectivity, we set σ_x_ and σ_y_ = 1.42, producing a more concentrated distribution of weights (See Fig. 2E). To match the average synaptic strength observed in the Felix implementation, we set k_C_ = 0.295, aligning the weight distribution across implementations while retaining spatially local inhibition.

This arrangement reflects the localised nature of inhibition in cortical circuits. Each excitatory neuron exerts its strongest influence on nearby inhibitory neurons, consistent with the principles of cortical feedback and lateral inhibition. The weights are initialised as before, decreasing smoothly with increasing distance according to the Gaussian profile. Additionally, excitatory-to-inhibitory connections have a delay of 0.5-time steps, corresponding to the model’s time resolution.

#### Inhibitory to excitatory neurons

For inhibitory-to-excitatory connectivity, a simple one-to-one connection scheme is used: each inhibitory neuron connects exclusively to its associated excitatory neuron at the same model location (i.e., at approximately micro-columnar scale). There is no probability distribution or weight initialisation involved in this setup, as the connection is deterministic (See Fig. 2F). The strength of the connection is uniform across all such pairs, reflecting the direct and localised nature of inhibitory feedback within cortical circuits. I-to-E connections have a delay of 0.5 time steps, corresponding to the model’s time resolution. The cumulative connection delay of E-to-I and I-to-E connections leads to the inhibitory circuit E-I-E operating at the model’s time resolution.

#### Excitatory to area-wide global inhibitory neurons

Each of the 625 excitatory neurons within a given cortical area projects to a single, area-specific global inhibitory neuron with a fixed synaptic strength of 1 and a delay of 0.5 time-steps. This global inhibitory neuron integrates the total excitatory activity within its respective area and provides uniform inhibitory feedback to all excitatory neurons within that region. This mechanism ensures network stability by preventing runaway excitation and maintaining balanced excitation-inhibition dynamics across the network.

#### Global inhibitory to excitatory neurons

The area-specific global inhibitory neuron provides feedback inhibition to all 625 excitatory neurons within its respective cortical area with a fixed synaptic strength and a delay of 0.5 time-steps. This ensures that the overall excitatory activity remains within physiological limits by dynamically modulating neural responses based on the cumulative activity within the area. Each excitatory neuron receives inhibitory input from the global inhibitory neuron, ensuring uniform regulation of excitation and preventing excessive firing.

### Inter-area connections

For the NEST implementation of the brain-constrained network we adopted the connectivity scheme used in earlier neuroanatomically informed simulations, ensuring that Felix and NEST were compared under an equivalent architecture. Specifically, the connectivity structure between cortical areas is guided by well-established neuroanatomical studies in primates and humans, ensuring biological plausibility. Adjacent areas within both perisylvian and extrasylvian regions are reciprocally connected, consistent with documented cortico-cortical pathways (Pandya and Yeterian 1985; Arikuni et al. 1988; Bressler et al. 1993; Dum and Strick 2005). Furthermore, multimodal connector hub areas (e.g., PB, PFi, AT, PFL) are linked through inter-area reciprocal connections, reflecting their role in integrating sensory-motor information across cortical domains (Fuster et al. 1985; Eacott and Gaffan 1992; Rilling et al. 2008; Petrides and Pandya 2009).

The inter-area, or between-area, connections in the model are exclusively E-E connections, ensuring that inter-area communication is mediated by excitatory connections. These between-area connections follow the same bivariate Gaussian spatial distribution used for local E-E connections (Eq. 7). However, the standard deviations (σ_x_ = σ_y_ = 9) are larger, reflecting the broader spatial spread of long-range cortico-cortical projections (See Fig. 2C). This adjustment accounts for the fact that between-area connections are more dispersed, enabling neurons to form links across a wider range of the target area.

In Felix, no distinction was made between within-system (black and blue arrows in Fig. 1) and cross-system (blue arrow with purple halo and purple arrows in Fig. 1) E-E connections; all inter-area connections were implemented with the same probability, regardless of whether they linked areas within the same functional system or across different systems. However, in NEST, we introduced a differentiation in connection probability due to fundamental differences in how excitation-inhibition interactions unfold over time in the two implementations: in Felix, excitation and inhibition interacted instantaneously without explicit delays, whereas NEST enforces synaptic delays, leading to stronger excitation and necessitating adjustments to limit cross-system activation.

Within-system connections refer to inter-area excitatory projections that remain within the same functional system – auditory, articulatory, visual, or motor – and these connections were kept identical to those in Felix. For example, in the auditory system, neurons in A1 connect to PB, and in the articulatory system, neurons in M1i connect to PFi with the same connection probability as in Felix. To reflect this, we set the scaling factor k_C_ = 0.13, ensuring that the probability distribution of these connections remains consistent with the original implementation (See Fig. 2H).

In contrast, cross-system connections, which link neurons across different functional systems (e.g., auditory-articulatory connections like PB to PFi, or auditory-motor connections like PB to M1L), were adjusted in NEST by reducing the probability of connection by half. This change reflects neuroanatomical findings showing that direct synaptic connectivity between different functional systems is sparser than within-system connectivity, often relying on additional relay areas for communication. To account for this, we set k_C_ = 0.065 for cross-system connections, ensuring that the NEST implementation better aligns with known cortical connectivity principles while introducing a biologically plausible differentiation that was absent in Felix.

On average, each excitatory neuron forms 33.78 connections (standard deviation: 0.15) with neurons within the same system and 16.65 connections (standard deviation: 0.01) with neurons from other systems (See Fig. 2G). If a connection is formed, the synaptic weight is initialised as a random value drawn from a uniform distribution between 0 and a predefined upper limit of 0.1 following the same rule of local connectivity as in Felix implementation. Additionally, all between-area connections have a fixed synaptic strength of 500 and a delay of 1 time-step (equivalent to twice the model’s resolution of 0.5 time-steps) (Table 3).

**Table 3:** Parameters for connections

Connection	Type	Boundaries	Probability	Initial Weights	Strength	Delay
Local						
E-E	Plastic	19 × 19	Gaussian Distribution (σ_x_ = σ_y_ = 3.2, *x̄* = ȳ = 0; q = 0, k_C_ = 0.15)	Uniform [0, 0.1]	500	1
E-I	Static	5 × 5	Deterministic = 1	Gaussian Distribution (σ_x_ = σ_y_ = 1.42, *x̄* = ȳ = 0; q = 0, k_C_ = 0.295)	0.295	0.5
I-E	Static	1 × 1	Deterministic = 1	1	500	0.5
E-GI	Static	625 × 1	Deterministic = 1	1	1	0.5
GI-E	Static	1 × 625	Deterministic = 1	1	65	0.5
Distant						
E-E (within system)	Plastic	19 × 19	Gaussian Distribution (σ_x_ = σ_y_ = 9, *x̄* = ȳ = 0; q = 0, k_C_ = 0.13)	Uniform [0, 0.1]	500	1
E-E (between system)	Plastic	19 × 19	Gaussian Distribution (σ_x_ = σ_y_ = 9, *x̄* = ȳ = 0; q = 0, k_C_ = 0.065)	Uniform [0, 0.1]	500	1

### Learning mechanisms

The Hebbian learning mechanism implemented in this model simulates established synaptic plasticity processes, specifically long-term potentiation and long-term depression, as described by Artola, Bröcher, and Singer (Artola and Singer 1993). This learning rule offers a biologically motivated approximation of experience-dependent neuronal plasticity. Importantly, in our model, only E-E neuron connections exhibit synaptic plasticity, whereas all other synaptic connections remain static.

The Hebbian learning rule encompasses both homo-synaptic and hetero-synaptic forms of plasticity, allowing for associative LTP and LTD. To operationalize this, we discretize the continuous range of possible synaptic changes into two fixed levels, **+**Δ and -Δ, where Δ is a small constant representing synaptic efficacy change. An excitatory synaptic connection from neuron *i* to neuron *j* is considered “active” if the estimated firing rate w_E_(*i*,*t*) of the presynaptic neuron *i* at time *t* exceeds a predefined threshold θ_pre_​. This threshold represents the minimum level of presynaptic activity required to induce LTP or homosynaptic LTD.

The modification of synaptic weight Δw(*i*,*t*) between a presynaptic neuron *i* and a postsynaptic neuron *j* is determined as follows:

8 $$\Delta w(i,j)=\left\{ \begin{array}{ll} +\Delta & \text{if } \omega_E(i,t)\ge \theta_{\mathrm{pre}} \text{ and } V(j,t)\ge \theta_{+}\;(\mathrm{LTP})\\ -\Delta & \text{if } \omega_E(i,t)\ge \theta_{\mathrm{pre}} \text{ and } \theta_{-}\le V(j,t)\le \theta_{+}\;(\text{homosynaptic LTD})\\ -\Delta & \text{if } \omega_E(i,t)< \theta_{\mathrm{pre}} \text{ and } V(j,t)\ge \theta_{+}\;(\text{heterosynaptic LTD})\\ 0 & \mathrm{otherwise} \end{array} \right.$$

where:

- θ_pre_: Threshold for presynaptic activity.
- θ_+_ and θ_−_: Upper and lower thresholds for postsynaptic membrane potential *V*(*j*,*t*).

The learning parameters were selected based on prior neurocomputational modelling studies (Garagnani et al. 2008; Garagnani and Pulvermüller 2016; Schomers et al. 2017; Tomasello et al. 2017, 2018) to ensure biologically plausible synaptic modifications relevant for language learning and associative memory. The Hebbian framework implemented in the model facilitates the formation of distributed cell assemblies (CAs), supporting the emergence of functional neural circuits responsive to language and sensory stimuli. Inhibitory connections remained static, ensuring consistent regulation of network activity by preventing excessive excitation.

While most learning parameters (See Table 4) were kept consistent across implementations, some adjustments were necessary to account for differences in how NEST and Felix handle spike propagation and synaptic updates. The lower postsynaptic threshold (θ_-_) was slightly reduced in NEST (0.11 vs. 0.14 in Felix). This adjustment was not required to prevent excessive synaptic strengthening in Felix but rather to accelerate the development of stable CAs. Using the same θ_-_ value across implementations resulted in stronger long-term depression (LTD) in Felix, which slowed down CA formation. Additionally, the learning rate (∆) was lower in NEST (0.0002) than in Felix (0.0008). Preliminary simulations indicated that using Felix’s learning rate in NEST led to the rapid formation of overly large CAs, suggesting that NEST facilitates faster associative learning and CA growth (see Supplementary Materials). Despite these parameter differences, both implementations successfully captured the key principles of Hebbian learning and distributed semantic representation (See Supplementary Materials for a more thorough parameter analysis).

**Table 4:** Parameters for E-E learning mechanisms

	Felix	NEST
θ_pre_	0.05	0.05
θ_+_	0.15	0.15
θ_-_	0.14	0.11
∆	0.0008	0.0002
Maximum value	0.225	0.225

To prevent runaway excitation and ensure that excitatory activity remains balanced within the network, a maximum synaptic weight of 0.225 was imposed on all plastic E-E connections. Since inhibitory connections remain static, an upper bound on excitatory synapses is necessary to prevent excessive potentiation, which could otherwise lead to network instability. Despite these parameter differences, both implementations successfully captured the key principles of Hebbian learning and distributed semantic representation.

### Spatial and temporal scales of the model

The general paradigm we have adopted in the present and previous works spans across several spatial and temporal scales, from single neurons to global cortical systems in space, and from fast firing patterns of neurons to global inter-area dynamics on a “behavioural scale”.

The functional base-units in the model are the local excitatory-inhibitory pairs of neurons in each area, where the excitatory neurons and their network of interconnections carry the core computational work (feature detection, learning and memory), and the inhibitory neurons serve sub-ordinate tasks, such as controlling network activity levels, sharpen feature tuning, or contributing to oscillations and their synchronisation (the latter two aspects do not play a role in the present work). We interpret each excitatory-inhibitory pair of neurons in the simulated grid as single representatives of clusters of excitatory and inhibitory cells, for example, in a micro-column, because neurons in small clusters typically act in a correlated way in the neocortex. In this sense, the model provides a spatially downsampled representation of cortex: many biological neurons are represented by a single model unit. A spiking excitatory neuron in the model therefore samples the typical activation pattern of excitatory neurons in a small neighbourhood (of the order of perhaps 100–200 μm in radius), and the corresponding local inhibitory cell represents the collective response of nearby interneurons (Fuster 1997; Braitenberg and Schüz 1998). Rather than simulating individual synapses, connections on this short scale are aggregated into effective coupling constants between exemplar units; the postsynaptic responses are therefore best understood as effective population-level PSPs, not unitary single-synapse PSPs (conceptually similar to downscaling approaches in large asynchronous networks; van Albada, Helias, & Diesmann, 2015). In addition, the cortex exhibits longer-range lateral connections between cortical pyramidal cells (which, for example, in the primary visual cortex predominantly connect neurons with similar orientation tuning properties (Gilbert and Wiesel 1989; Hubel 1995). The probability of these connections decays quickly with distance on a scale of 5–10 mm. It is this sort of connection that the Gaussian coupling kernels in our model implement. Therefore, each grid-cell in the model can be interpreted as a cortical patch of a few hundred micrometres in diameter, with the activity of the associated model neuron reflecting the typical firing pattern of neurons within such a patch, whereas the connections made from any specific location fan out a few millimetres. Space in the model does therefore not linearly map to space in a real cortical area but only preserves topology. For this reason, we mostly refer to “cells” and “units” in this paper but do not often use physical length units.

The fast neuronal time scale of the model is anchored to the millisecond range: in both Felix and NEST a single excitatory update step corresponds to 1 ms (see Numerical Simulation Details). Given this choice of microscopic time scale and the size of the network, the overall temporal structure of our simulations becomes effectively compressed: stimuli are presented only for a few time steps (i.e. a few milliseconds), and learning unfolds over hundreds to thousands of such steps, which should be understood as an abstract approximation of much longer behavioural and developmental time scales. This limitation is acceptable for the present purpose of comparing Felix and NEST and modelling relative timing between cortical areas; however, more realistic studies of microcircuits or behaviour would require longer stimulation durations and explicit modelling of slower plasticity processes.

Finally, it may seem inconsistent that we use spiking neurons for the excitatory cells and graded neurons for the local inhibition. This is motivated by the fact that the I-cells in the model basically only regulate the activity locally, whereas the E-cells implement the Hebbian learning dynamics and “information processing”. We have also run simulations in which the inhibitory neurons were implemented as spiking units. These tests produced promising results and reproduced the same qualitative network, learning, and retrieval properties. However, because the original Felix model implement local inhibition as a mean-field pool, we retain the graded (mean-field) inhibitory units in the present study for comparability.

Whereas the above constraints take some of the physiological “realism” of the model, many of them are not crucial as we see very similar activation, learning and retrieval patterns under a wide range of parameters. One crucial aspect may be the short stimulus times as they limit the interpretability of spiking activity in the model. However, we see similar excitation-inhibition cycles/oscillations as in the model also in earlier, simpler models with on-going stimulation (Wennekers and Sommer 1999; Bibbig et al. 2002). This, however, requires further study and is out of the scope of the present work. Currently, it is not possible to simulate every desirable feature in reasonable time on available hardware. However, for specific purposes, the model could be quite easily adapted using the new NEST implementation.

### Numerical simulation details

In Felix, inhibitory neurons were updated at each simulation time step (t) based on excitatory input from the previous time step (t − 1), while excitatory neurons were updated within the same step (t) using inhibition computed during the same step. This allowed for immediate interaction between excitation and inhibition without requiring explicit synaptic delays. Felix therefore operated with a simulation resolution of 1.0 units per time step (corresponding to 1 millisecond).

In contrast, NEST requires all synaptic delays to be explicitly defined and enforces a minimal non-zero delay. To accommodate this, we set the internal simulation resolution to 0.5 ms per computational step in NEST, allowing inhibitory and excitatory updates to occur on a finer temporal grid. However, the effective excitatory connection delay remains 1 ms (two internal steps), matching the timing of the original Felix implementation (see Connectivity). Accordingly, throughout the present work we report all timing in excitatory time steps, corresponding to 1 ms in both Felix and NEST. To maintain comparable neural dynamics across both implementations, we adjusted synaptic delays accordingly (see ‘Connectivity’) and doubled all membrane and adaptation time constants (τ, τ_ADAPT_) in NEST. While the integration step and synaptic delays are thus defined in milliseconds, the durations of external stimuli and of learning protocols in our simulations should be regarded as a temporally compressed approximation of much longer behavioural time scales.

The Felix implementation updates all leaky state variables (e.g., membrane potential, adaptation currents) using the forward Euler method, where values are incrementally adjusted at each fixed time step. In contrast, the NEST simulator solves the underlying linear differential equations with an exact integration scheme (Rotter and Diesmann 1999). This method assumes a constant input over the integration step and uses a pre-computed exponential decay factor, allowing for a numerically stable and efficient update via a single multiply-and-add operation. These differences in numerical integration are a likely source of any subtle variations observed in the dynamics between the two simulations.

### Model comparisons

To document differences and similarities between the model implementations in NEST and Felix from the neuron level to whole model dynamics, we have conducted different simulations with increasing levels of complexity: (i) a single excitatory neuron and (ii) 12 areas with all model features as described in previous sections after training (See next section). This allows us to report, in order, (i) the cell membrane potential and spiking behaviour over time during and after a spike train input and (ii) emergent whole-network spatiotemporal dynamics of spiking, as well as CA formation.

### Single neuron behaviour

To assess the consistency of neuronal dynamics across implementations, we conducted simulations in both NEST and Felix, focusing on a minimal network consisting of a single excitatory and a single inhibitory neuron. The excitatory neuron was stimulated using an external constant current applied for a duration of 16 time steps. Throughout the simulation, we measured the membrane potential of the inhibitory neuron and excitatory neuron along with its adaptation and spiking activity. These recorded variables were used to evaluate the alignment of neuronal behaviour between the two implementations.

### Multi-area model – training procedure

We replicated the multi-area object-and-action word-learning model originally run in the Felix simulator in both the Felix and NEST environments. To capture network-to‐network variability, we generated 12 independent network simulations, each using a different random seed to stochastically wire excitatory-to-excitatory connections and generate random input patterns. During learning, these external input patterns co-activated targeted subsets of neurons and drove Hebbian plasticity of the E→E synapses. Our plasticity rule combined LTP – strengthening of synapses between co-active neurons in accordance with the principle “neurons that fire together wire together” (Hebb 1949), LTD – the weakening of synapses between neurons that were not co-active, “neurons out of sync delink” (Artola and Singer 1993). After repeated presentations, neurons that regularly fired together became strongly interconnected, forming characteristic “cell-assembly circuits” (Hebb 1949).

Prior to simulation, 12 stimulus triplets were created, each consisting of 20 randomly selected excitatory neurons in three of the four primary cortical areas (A1, M1i, V1, or M1L). Each learning trial comprised 16 time steps. At each time step, the triplet’s neurons were activated within those three areas, while the fourth received uncorrelated random input. This setup produced sparse activation, engaging about 3% of neurons in each 25 × 25 primary area.

Stimulation was applied simultaneously to the primary auditory cortex (A1) and inferior motor cortex (M1i), along with either the primary visual cortex (V1) (for object-related words) or lateral motor cortex (M1L) (for action-related signs). This setup mimics natural word learning, where a novel word form is uttered while an object is visually perceived (Vouloumanos and Werker 2009) or while performing a relevant motor action (Tomasello and Kruger 1992). To reflect natural learning variability, stimulation patterns included uncorrelated noise in V1 for action-related words and in M1L for object-related words. This ensured that while the correlation between word form and semantic features was high in the relevant modality, it remained low in the non-relevant one. The patterns applied to visual and motor cortices captured the experiential and environmental context associated with learning word meanings – representing, for instance, the object or action a word refers to.

Each network learned 12 distinct triplets of sensory-motor activation patterns, simulating the learning of six action-related signs and six object-related signs. White noise was continuously presented to all model areas throughout the learning phase, regardless of whether stimulation patterns were applied, maintaining baseline neural activity. Following the 16 time step stimulation period, a stimulus-to-stimulus interval (STSI) was introduced, during which no input was given to the model. This interval lasted until network activity returned to baseline levels, allowing global inhibition in multimodal hub areas (PFi and PB) to reset before the next trial. This ensured that consecutive learning trials did not interfere with one another.

Each word was trained for 2000 trials, resulting in a total of 24,000 trials per network across the 12 network simulation runs, amounting to 288,000 learning trials in total. This number of repetitions was chosen based on extensive previous work, which demonstrated stabilisation of learning outcomes after 1000 exposures per words (Garagnani et al. 2008; Schomers et al. 2017).

### Evaluation

After the learning phase, we examined the neurophysiological mechanisms underlying object- and action-word processing across different network architectures. To assess how learned representations were encoded within the network, each of the twelve trained acoustic patterns was presented to the primary auditory cortex (A1) and in different trials twelve motor patterns were presented to the primary articulatory area (M1i). The resulting neural activity was mapped across all modelled cortical areas.

*Mapping of cell assembly circuits* Each evaluation trial began with a baseline period of 5 time steps without external input, followed by 2 time steps of pattern presentation to A1, simulating speech comprehension. The activation was then allowed to propagate through the network for 60 time steps (Garagnani et al. 2008; Schomers et al. 2017; Tomasello et al. 2017; Tomasello et al. 2018). Throughout this process, all neurons received white noise, consistent with the learning phase. To evaluate network activity, each excitatory neuron was assessed based on its spiking behaviour. A neuron was considered active if it fired at least once during an evaluation trial. For each learned pattern, three trials were conducted where the corresponding auditory stimulus was presented to A1, and the active neurons were recorded. In a separate set of trials, the same procedure was repeated with stimulation in M1i to simulate speech production. This process was also repeated three times. A neuron was classified as part of a cell assembly for a given pattern if it spiked at least four times across the six trials (three auditory and three articulatory stimulations). This procedure was applied consistently across all networks and averaged for different word types to ensure robust identification of cell assemblies for both NEST and Felix implementations.

### Statistical analysis

A statistical analysis was conducted to examine differences in cell assembly size and distributions between word-related categories across different network areas for each implementation. A two-way repeated-measures ANOVA was performed with the factors WordType (2 levels: Action and Object) and Areas (12 levels: A1, AB, PB, PFi, PMi, M1i, V1, TO, AT, PFL, PML, M1L). In the case of a significant interaction effect, a post-hoc Tukey test was conducted for the factor WordType within each area, with Bonferroni correction applied to adjust for multiple comparisons. Importantly, no direct statistical comparisons were made between implementations, as the objective was to assess whether similar overall patterns emerged rather than to establish a precise one-to-one correspondence between them. The focus of this analysis was to observe converging trends in the effects of WordType on cell assembly size across different implementations, rather than evaluating their exact equivalence.

### Simulation framework and code availability

All simulations were conducted using NEST 3.6, extended with the **felixmodule** package, developed as part of the present work, which provides custom neuron models (felix_exc, felix_inh), a synapse model (abs_synapse), and a recording device (felix_spike_recorder). The full implementation, including network definitions, connectivity parameters, plasticity rules, and simulation scripts, is publicly available on GitHub at https://github.com/MaximeCarriere/cogninest. This ensures full transparency and reproducibility, allowing other researchers to replicate, modify, and extend our work for further investigations in neurocomputational modelling. Simulations were executed on the HPC cluster of Freie Universität Berlin, using 6 CPU cores of Intel Xeon processors under AlmaLinux 8 with the Slurm batch system (Bennett et al. 2020).

## Results

### One neuron comparison

**Fig. 3:**
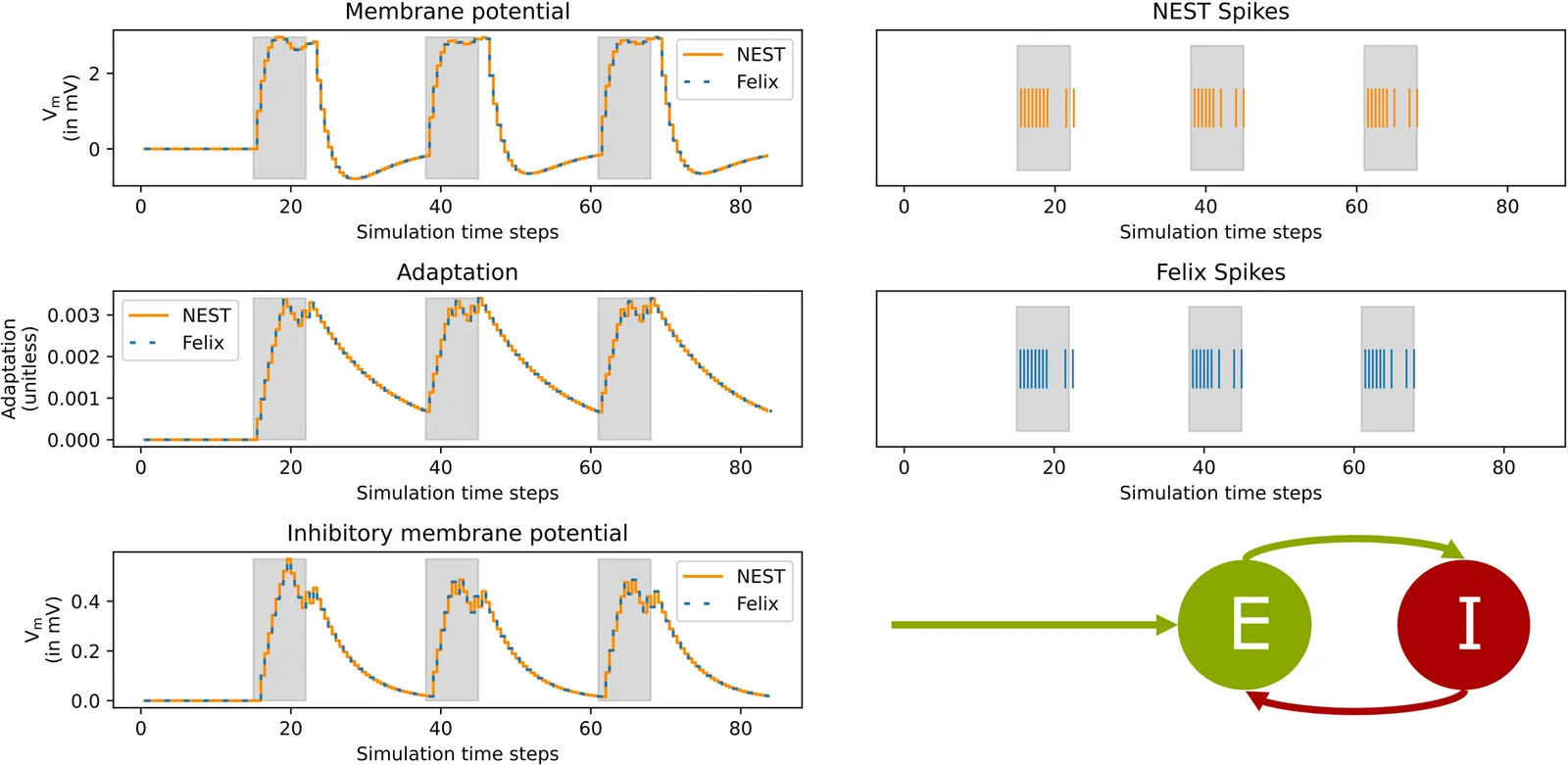
Comparison of neuronal dynamics between NEST and Felix implementations. The left panels display the membrane potential (top), neuronal adaptation (middle) and inhibitory membrane potential (bottom) of a pair of a single excitatory neuron and a single inhibitory neuron over time in both NEST (orange) and Felix (dashed blue). The right panels show the corresponding spiking activity for the same neuron in NEST (top) and Felix (bottom), where each vertical line represents a spike event. The shaded grey regions indicate periods of external stimulation. The solid NEST traces and dashed Felix traces overlap perfectly, indicating a match between the two implementations

To validate the consistency of neuronal dynamics across implementations, we analysed the membrane potential, adaptation, and spiking behaviour of a minimalist pair of a single excitatory and a single inhibitory neuron in both NEST and Felix by stimulating the excitatory cells with an external constant current of 16 time-steps and recorded its membrane potential, adaptation and spikes (Fig. 3). The results demonstrate a perfect match in the evolution of membrane potential over time, with identical depolarisation and repolarisation cycles observed in both implementations. During periods of external stimulation (shaded grey regions in Fig. 3), neurons in both models exhibited similar activation, with membrane potentials rising to the threshold, followed by sharp resets corresponding to spike events. Similarly, the neuronal adaptation variable followed an identical trajectory across implementations. The gradual increase in adaptation during periods of high firing activity, followed by a slow decay after stimulation, was identical between NEST and Felix, confirming that the adaptation mechanism was preserved across both platforms. This ensures that neurons in both implementations exhibit the same history-dependent modulation of excitability and inhibitory mechanisms.

The spiking patterns further confirmed the equivalence between NEST and Felix at the neuronal level. The timing and number of spikes were precisely aligned in both implementations, indicating that the underlying integrate-and-fire mechanism and spike thresholding processes operate consistently across both platforms. This finding is critical, as even small discrepancies in spike generation could lead to divergent network-level behaviour. Overall, these results confirm that the fundamental neuronal properties, membrane dynamics, adaptation, and spiking, are faithfully replicated in NEST. Therefore, any differences observed at the level of larger-scale network dynamics, such as cell assembly formation, inter-area propagation, or temporal activation patterns, can be attributed to system-level mechanisms (e.g., synaptic delays, connectivity patterns), rather than inconsistencies in the basic neuronal model.

### Cell assemblies size and topographical distribution: Felix vs. NEST

**Fig. 4:**
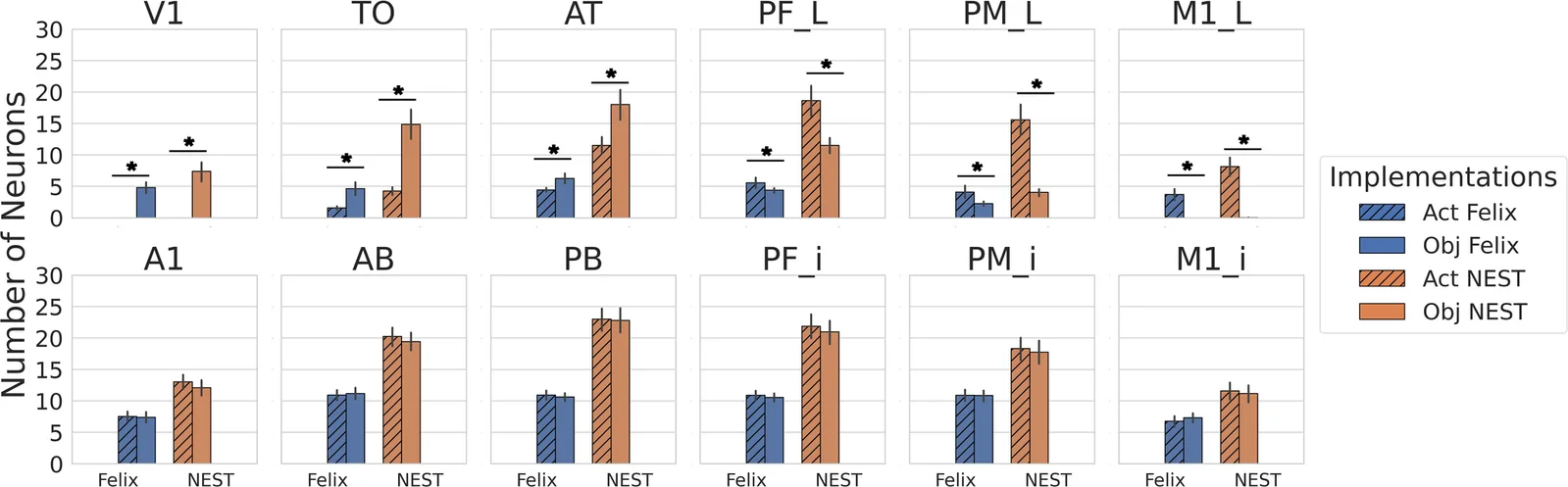
Comparison of Cell Assembly (CA) distributions across NEST and Felix implementations. The mean number of CA neurons is plotted for each cortical area. Blue bars represent Felix, orange bars represent NEST. Within each implementation, hatched bars show action words and solid bars show object words. Notable topographical differences emerge in extrasylvian regions, particularly in the lateral motor cortex (M1L), where action words elicit the highest CA neuron counts, and in the primary visual cortex (V1), where object words show the strongest activation. These patterns are consistent across both model implementations

The distribution of cell assemblies (CAs) across the network reveals a qualitative similarity between the NEST and Felix implementations (Fig. 4). Across all modelled cortical areas, CA formation followed the same topographical pattern in both implementations, indicating that the transition to NEST preserves the general organisation of semantic representations. Quantitatively, however, the size of CAs differed substantially, with NEST producing assemblies roughly twice as large as those in Felix. This difference in CA size is analysed in detail in the discussion section. Despite these quantitative differences, the overall network organisation and activation patterns remained comparable across implementations, supporting the conclusion that NEST maintains the main functional properties of the original model.

Crucially, the emergence of semantic specificity is consistent across both implementations. Action-word CAs are more prevalent in the motor system, particularly in M1L, PML and PFL whereas object-word CAs are more dominant in the visual system, especially in V1, TO and AT. Additionally, action words showed only limited emergence in the visual system, with no CA neurons present in V1, while object words exhibited only limited emergence in the motor system, with no CA neurons found in M1L. This further reinforces the specialisation of sensorimotor areas for conceptual grounding, while phonological representations remain largely uniform across word types in the perisylvian regions (A1, AB, PB, and articulatory motor areas M1i, PMi, PFi), where no word-type-related differences were observed in either implementation. Note that in both implementations the multimodal hub areas (PB, PFi, AT, and PFL) contain a higher number of CA cells compared to the secondary areas (AB, PMi, TO, PML), which in turn have more CA cells than the primary areas (A1, M1i, V1, M1L). This pattern appears to be independent of whether the represented word is object- or action-related.

The descriptions above were confirmed by the two-way repeated-measures ANOVA (WordType: 2 levels X Area:12 levels) conducted on topographical CA size and distribution in both Felix and NEST Model output data.

For Felix, the analysis revealed a significant main effect of Area, (F(11, 121) = 198.04, *p* < 0.001), indicating that cell assembly size varied significantly across different brain regions. However, the main effect of WordType was not significant, (F(1, 11) = 0.35, *p* = 0.565), suggesting that WordType alone did not influence cell assembly size across all areas. Importantly, a significant interaction between Area and WordType, (F(11, 121) = 25.41, *p* < 0.001), indicated that the effect of WordType on cell assembly size varied by cortical areas.

Post-hoc Tukey tests with Bonferroni correction (12 comparisons; critical p-value = 0.0042) revealed that Action words elicited significantly larger cell assemblies in motor-related areas, whereas Object words elicited significantly larger cell assemblies in visual-related areas. Specifically, significant differences in cell assembly size between Object and Action word types were observed in all extrasylvian regions (*p* < 0.0042). In contrast, no significant differences between Action and Object word types were found in any perisylvian regions.

For NEST, a similar two-way repeated-measures ANOVA confirmed a significant main effect of Area, (F(11, 121) = 587.23, *p* < 0.001), further demonstrating that cell assembly size varied across brain regions. The main effect of WordType was non-significant (F(1, 11) = 0.36, *p* = 0.563), indicating that WordType alone did not influence cell assembly size when all areas were considered together. However, there was a strong significant interaction between WordType and Area, (F(11, 121) = 137.68, *p* < 0.001), confirming that the effect of WordType on cell assembly size was region-dependent.

Post-hoc Tukey tests with Bonferroni correction revealed the same pattern observed in Felix, where Action words led to significantly larger cell assemblies in motor-related regions, while Object words led to significantly larger cell assemblies in visual-related regions (p-value < 0.0042). These effects were limited to extrasylvian regions, while no significant differences were found in perisylvian regions.

Overall, the preservation of semantic specialisation and cortical distribution in NEST confirms the model’s robustness and reproducibility, while the observed increase in CA size highlights a quantitative difference without altering the fundamental computational structure underlying the network.

### Cell assemblies emergence over learning episodes: Felix vs. NEST

**Fig. 5:**
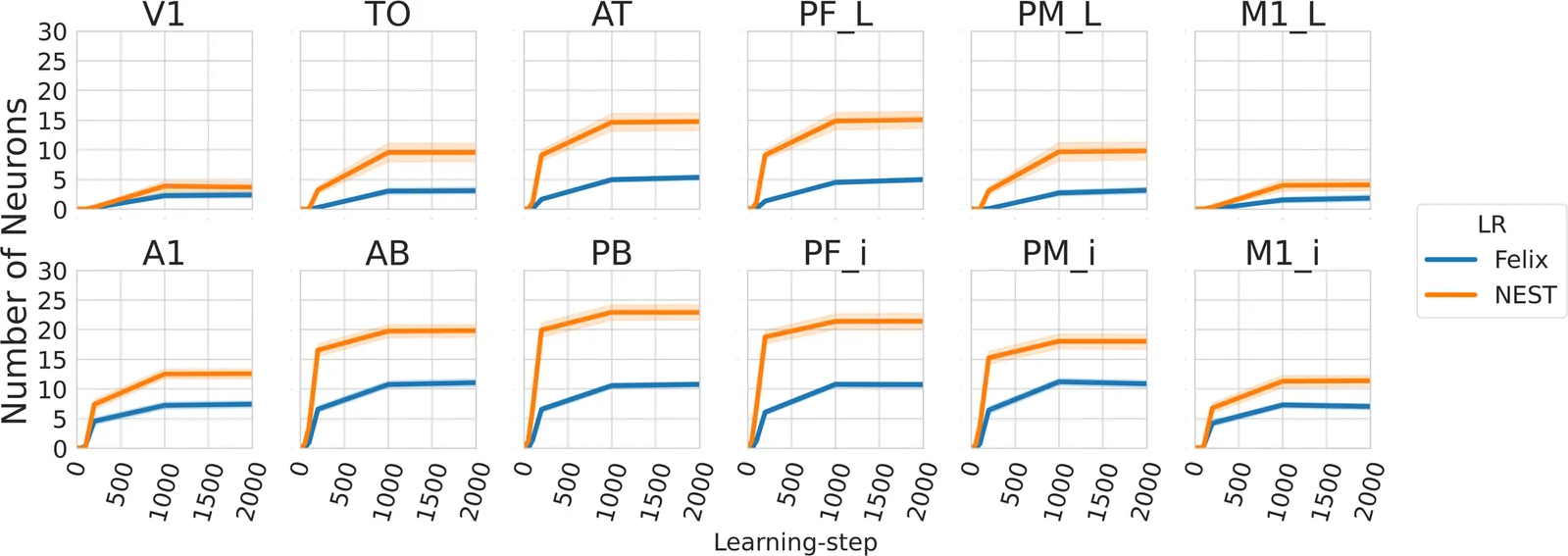
Evolution of Cell Assembly Size counted as the average number of CA neurons per area across Learning-steps in NEST and Felix Implementations. The mean number of neurons per cell assembly (CA) is plotted for each cortical area over the course of learning episodes in both NEST (orange) and Felix (blue) implementations. Each panel represents a different cortical area, showing the progressive increase in CA size as learning progresses

The development of cell assemblies (CAs) over the course of learning followed a similar trajectory in both NEST and Felix implementations, with a progressive increase in CA size across all cortical areas as learning progressed (Fig. 5). However, a consistent and substantial difference emerged between the two implementations: CAs formed in NEST were systematically larger than those in Felix across all modelled regions, confirming the earlier observations. This difference was evident from the earliest stages of learning and remained stable as learning progressed.

Across all perisylvian areas (A1, AB, PB, PFi, PMi, M1i), CA growth was steepest in the first 500 learning steps, after which CA sizes stabilised around 1000 learning steps, indicating a consolidation of network learning. The same stabilisation was observed in extrasylvian areas, including motor regions (M1L, PML, PFL) for action words and visual regions (V1, TO) for object words, reinforcing the semantic specificity of CA distributions. This plateau in CA growth suggests that, beyond this point, no further large-scale reorganisation occurs, and the network has reached a stable configuration for encoding learned representations.

Importantly, the multimodal connector hub areas (PB, PFi, AT and PF_M_) also showed a clear difference in CA sizes between the two implementations. While these areas were strongly engaged in both models, NEST exhibited a more pronounced increase in CA size, consistent with its greater recruitment of neurons.

Overall, these results confirm that the transition from Felix to NEST preserves the core learning dynamics and topographical organisation of CAs. While NEST consistently forms larger CAs, the trajectory of their growth, stabilisation after 1000 learning steps, and semantic distribution remain unchanged, reinforcing the robustness of the model across implementations.

### Spatiotemporal activation: Felix vs. NEST

**Fig. 6:**
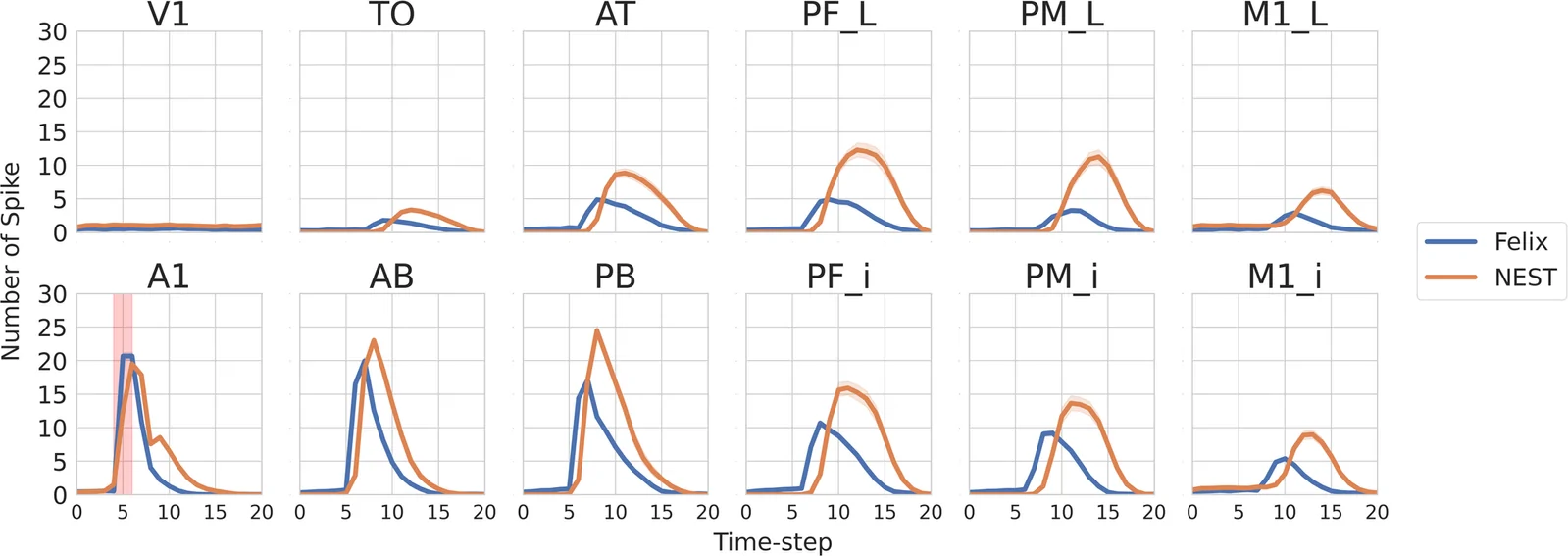
Time course of activation following auditory (A1) stimulation for an action word across cortical areas in NEST and Felix implementations. The number of spikes is plotted over simulation time steps for each cortical area, separately for NEST (orange) and Felix (blue). One simulation time step corresponds to 1 ms of model time. The stimulation period in A1 (2 time steps, i.e. 2 ms on the microscopic scale) is indicated by the shaded red area. Activity spreads from A1 to perisylvian regions (AB, PB, PFi, PMi, M1i) and further into extrasylvian lateral prefrontal and sensorimotor regions (PFL, PML, M1L) in both implementations. The overall duration of the stimulation and response should be interpreted as a temporally compressed, schematic representation of a much longer real cortical process (see “Spatial and Temporal Scales of the Model”)

To investigate the temporal evolution of activation, we analysed the spiking activity across cortical areas following auditory (A1) stimulation for an action word in both NEST and Felix implementations (Fig. 6). The stimulation protocol included an initial 5 time steps baseline period during which no external input was applied, ensuring that spontaneous network activity remained at minimal levels. This was followed by 2 time steps of direct stimulation to A1, which led to a rapid increase in neuronal activity in this area before activation propagated throughout the network.

Following stimulation, activation quickly spread from A1 to adjacent perisylvian regions, including secondary auditory areas (AB, PB) and articulatory motor regions (M1i, PMi, PFi). This early propagation phase was nearly identical in both implementations. However, as activation reached multimodal regions, key differences emerged: in NEST, activation levels were consistently higher, particularly in prefrontal (PFi), premotor (PML), and primary motor (M1L) areas. In contrast, Felix exhibited lower peak activity, with stronger engagement remaining largely confined to perisylvian regions. Importantly, both implementations demonstrated clear category specificity, with strongest activation in the motor system and very low in the visual system. In both models, no activation was observed in V1, reinforcing the expected semantic category specificity – that is, action words preferentially engage the motor system rather than visual processing areas.

A key distinction between NEST and Felix arises due to synaptic delays in NEST, which were not present in Felix. In Felix, activity in excitatory neurons was updated within the same time step as inhibition, allowing for near-instantaneous transmission of activation between neurons within a single area. However, NEST requires explicit synaptic delays, resulting in a gradual prolongation in the timing of activation spread. This effect becomes more pronounced as activity propagates further from the initial stimulation site, leading to increasingly delayed peak activations in distant regions such as M1L and PML compared to Felix. The impact of this delay accumulates along the processing hierarchy, such that regions further from A1 exhibit peak activity at progressively later time steps in NEST relative to Felix.

A similar analysis was conducted for object words, revealing stronger visual system activation (particularly in V1 and TO) compared to the motor system. These results, available in the supplementary material, further support the model’s ability to capture semantic category specificity, with motor areas preferentially activated for action words and visual areas for object words. Thus, the time-course activation results validate the observed differences in cell assembly size, demonstrating stronger motor activation for action words and stronger visual activation for object words. These activation patterns also confirm the systematic increase in CA size in NEST compared to Felix.

Importantly, the spatiotemporal activation profiles obtained in both implementations align with neurophysiological evidence: auditory regions show an early, strong response at the stimulation site (mimicking auditory perception), followed by more moderate, modality-specific cortical recruitment of extrasylvian regions distal to the stimulated region during word recognition processes (Hauk et al. 2004; Pulvermüller 2005). The NEST simulations, which include explicit conduction delays, reproduce a slightly more gradual propagation of activity across areas, providing a testable plausible temporal pattern and suggesting that such delays may contribute to the graded and stronger recruitment of neural matters across the modelled regions.

## Discussion

In this study, we implemented the 12-area spiking Brain-constrained Perception Action Model of Cognition and Language (BPAM-o-CAL) within the NEST simulation framework, ensuring greater accessibility, reproducibility, and scalability compared to the previously used Felix package. The model consists of 12 interconnected cortical areas, incorporating spiking neurons with inhibitory populations, enabling biologically plausible neural dynamics and cell assembly formation for concept, symbol, and semantic processing. While maintaining identical network architectures, synaptic organisation, and learning mechanisms across implementations, we examined the emergence of cell assemblies and their distributions across cortical areas following Hebbian-based associative learning of action and object words in action and perception system. The current work should thus be regarded as a first-stage replication aimed at faithfully transferring and validating the well-established Felix model within NEST before introducing further biological refinements. Importantly, the present simulation serves as proof that the core results previously obtained with Felix can be reproduced in a different simulation environment, requiring only minimal adjustments. This step ensures full functional equivalence and provides a solid foundation for future developments, incorporating, for instance, anatomical scaling, realistic delays, and inhibitory plasticity.

Although the primary goal was a one-to-one numerical replication between Felix and NEST, differences in the simulation engines posed significant challenges. A felix-package had to be implemented to extend NEST and allow it to cover the neuron-types and learning rule used. The most critical difference involved synaptic delays, which are biologically plausible and required in NEST but are absent in Felix. In Felix, inhibition was instantaneously computed alongside excitation and external input. In contrast, NEST enforces a minimal synaptic delay, requiring excitatory neurons to process inhibitory inputs from the previous time step. This fixed delay introduces a more realistic temporal separation between excitation and inhibition, consistent with the presence of synaptic and axonal conduction delays in the brain (Waxman 1980; Sabatini and Regehr 1996; Swadlow 2000).

The inclusion of synaptic delays in NEST resulted in excitatory neurons remaining active for longer periods before inhibitory regulation became effective. The relatively later onset of inhibition leads to less inhibition being available at early time steps, shortly after stimulation. As a consequence, excitatory activity in NEST could spread more widely and recruit additional neurons before delayed inhibition became effective. This mechanism explains both the larger activation peaks and the slight temporal shift of CA activity observed across several cortical areas (Fig. 6). The resulting increase in overall excitation produced systematically larger cell assemblies – about twice as many neurons per assembly as in Felix (Fig. 4). Further analyses confirmed that this effect reflected enhanced network excitation rather than greater learning efficiency (Supplementary Figs. S1, S2). Such excessive excitation disrupted the excitatory–inhibitory balance essential for stable cortical processing (Wilson and Cowan 1973; van Vreeswijk and Sompolinsky 1996; Sadeh et al. 2015; Denève and Machens 2016), occasionally leading to merging of cell assemblies. Because single-neuron parameters (membrane potential dynamics, thresholds, adaptation) were identical in both implementations (Fig. 3), the altered activation patterns can be attributed solely to the delayed timing of synaptic inhibition in NEST (Figs. 4 and 6).

To address excessive excitation, we introduced distinct connectivity constraints differentiating functional systems by within-system and cross-system inter-area connections. Cross-system connectivity was reduced by half, while within-system connections remained unchanged from the Felix model. This adjustment mitigated runaway excitation, stabilised the network, and restored functional specificity. Furthermore, this modification aligns with neuroanatomical evidence suggesting weaker cross-system compared to within-system connectivity (Passingham et al. 2002; Markov et al. 2013).

Despite these adjustments and resultant differences, the NEST implementation faithfully replicated core neuronal properties observed in Felix, including membrane potential dynamics, adaptation, and spiking behaviour, confirming robustness across simulation platforms. Both implementations successfully reproduced the semantic category-specificity of CAs, with action words primarily engaging motor areas (M1L, PML, PFi) and object words strongly activating visual regions (V1, TO, AT), consistent with sensorimotor theories of semantic representation (Pulvermüller 1999; Hauk et al. 2004; Pulvermüller et al. 2009; Shtyrov et al. 2014; Kemmerer 2015). Notably, perisylvian regions (A1, AB, PB, M1i, PMi, PFi) did not show word-type-related differences, reinforcing the distinction between phonological processing and conceptual grounding (Pulvermüller 2018). The fact that these patterns previously described in Felix-based simulations (see for a detail discussion Tomasello et al. 2018) similarly emerged in NEST shows topographical distinct semantic circuits for different word types and are to a degree resilient against variations in spike timing. These findings provide further computational validation of neurobiologically-founded models of language in which word meaning is distributed across sensorimotor and multimodal integration hubs rather than being localised in a single amodal region (Binder et al. 2011; Pulvermüller 2013).

Furthermore, the implementation in NEST significantly enhanced computational efficiency, reducing simulation runtime nearly sixfold compared to Felix. This substantial improvement in computational speed is particularly critical for future expansions of the model, such as incorporating additional cortical regions or including the right hemisphere, which would demand increased computational demands. Beyond replicating previous results, implementing BPAM-o-CAL in NEST offers significant practical advantages. Unlike Felix, which relied on outdated, unsupported graphics libraries (XView) incompatible with modern 64-bit systems, the NEST-based model utilizes a contemporary simulation environment accessible through a Python interface. Additionally, encapsulating the model within a Docker image allows seamless installation and enhanced reproducibility across platforms, facilitating future research and model extensions.

## Implications and future directions

We report successful replication of key neurophysiological signatures of single cell and circuit level activity in the Felix and NEST implementations of the 12-area BPAM-o-CAL model. These results demonstrate the potential of the implementation in NEST to become a scalable, open-source option for large-scale brain-constrained neural modelling. Such an implementation would be useful for several reasons. First, the Felix implementation currently only offers simulations of representational learning with rather small numbers of different representations. A substantial increase of network size would be required to implement, for example, large vocabularies or rich repertoires of concepts. Furthermore, syntactic learning requires the availability of a large stock of symbols, so that the new tool could also assist in modelling aspects of grammar. Likewise, for the modelling of additional cognitive and perceptual brain systems and related capabilities, it may be necessary to add implementations of the dorsal visual pathway, the somatosensory system, olfactory and gustatory systems and multimodal areas in the parietal and prefrontal cortex, or emotion-related regions (e.g., limbic structures, insula), which are known to play a role in affective and social aspects of communication (Moseley et al. 2012; Vigliocco et al. 2014; Hinojosa et al. 2020). Such extensions would require a substantial increase in computational resources, which we could not achieve in the past based on Felix implementations and parallel extensions thereof. We hope that the computational infrastructure of NEST will allow us to realize at least some of these projects.

Future work should also explore alternative plasticity rules, reinforcement learning mechanisms, and multimodal sensory integration within this framework to refine computational models of language learning and representation. One promising direction is the incorporation of dendritic computations, which have been shown to play a crucial role in sequence detection and temporal processing (Hawkins and Ahmad 2016; Quaresima et al. 2023). Given that syntax relies on the precise encoding of word order and hierarchical structure, integrating dendritic nonlinearities into our spiking model could provide a mechanistic account of syntactic processing in biologically plausible circuits (Pulvermüller 2003). In addition, future work should address the current simplification of using equal numbers of excitatory and inhibitory neurons. Adjusting this ratio toward a more biologically plausible 4:1 proportion will enhance the neurophysiological realism of the model, though it requires revising the local inhibitory connectivity scheme (currently one-to-one). This will be considerably easier to implement in NEST than in the Felix framework, as the first starts from single neurons and synapses that can be connected without further constraints, whereas the latter starts from less flexible two-dimensional arrays of cells (fields). Likewise, introducing plastic connections for inhibitory neurons and additional adaptive mechanisms, such as neuron-intrinsic or homeostatic plasticity, may be required to allow the model to maintain stable firing dynamics in the presence of ongoing synaptic plasticity. Including these mechanisms would also enhance the models’ biological realism (Lamsa et al. 2005).

The NEST framework already provides access to a variety of learning rules, such as spike-timing–dependent plasticity (STDP; Song et al. 2000; Morrison et al. 2007) which could be systematically tested within our architecture. Comparing the behaviour of the model under different synaptic learning mechanisms would offer valuable insight into how plasticity dynamics influence associative learning, stability, and cell assembly formation in large-scale language networks.The modularity of NEST makes such extensions feasible, paving the way for more accurate simulations of cortical microcircuit organisation.

Another promising extension would be to incorporate distance-dependent conduction delays between cortical areas. Adding inter-area delay variability could help examine how transmission times influence synchronisation, oscillatory coupling, and temporal integration in language-related networks. This adjustment would increase the temporal realism of the model and allow to study the spatio-temporal dynamics of cortical representations in much more detail than possible in our previous works. That said, greater biological realism does not necessarily guarantee a better mechanistic explanation. Future studies are therefore needed to directly test how much anatomical and temporal detail is truly required for explanatory validity, and whether more abstract, but still biologically plausible, models may, in some cases, capture the essential mechanisms equally well. By leveraging these model refinements, we can make the network more neurophysiologically realistic while simultaneously expanding its scope to investigate key questions in language structure and grammar emergence. With enhanced scalability, computational efficiency, and open-source availability, these advancements will not only accelerate research in neural language processing but also contribute to broader investigations in cognitive neuroscience, artificial intelligence, and neuromorphic computing. By making the implementation publicly available, other research teams worldwide will be able to test their own hypotheses and extend the model for new applications. This fosters a collaborative scientific effort, where different computational neuroscience groups can explore alternative architectures, learning paradigms, or even integrate multimodal sensory input (Plesser et al., 2025).

## Conclusion

By transitioning to NEST, we established an accessible, scalable framework for brain-constrained modelling of language and cognition: the 12-area BPAM-o-CAL network. The new implementation successfully replicated key features of the original Felix model, including biologically realistic spiking behaviour, homeostatic balance between excitation and inhibition, Hebbian-based associative learning, and the emergence of distributed, semantically meaningful cell assemblies organised in a topographical manner. These assemblies displayed sensorimotor specificity – differentiating between action- and object-related words – and maintained phonological invariance in perisylvian areas, consistent with previous findings. At both the cellular and network levels, the successful replication of neurophysiological properties confirms the robustness of the new implementation, validating NEST as a robust simulation environment for investigating neural mechanisms underlying language and cognition. Beyond replication, the new implementation enables substantial model extensions. Improved computational efficiency and open-source availability allow scaling to larger vocabularies and more complex conceptual structures, opening new avenues for exploring symbolic, syntactic, and multimodal processes in biologically grounded neural models of language (Plesser et al. 2025).

## Supplementary Information

Below is the link to the electronic supplementary material.

Supplementary Material 1

## Data Availability

All simulations were conducted using NEST 3.6, extended with the felixmodule package, developed as part of the present work, which provides custom neuron models (felix_exc, felix_inh), a synapse model (abs_synapse), and a recording device (felix_spike_recorder). The full implementation, including network definitions, connectivity parameters, plasticity rules, and simulation scripts, is publicly available on GitHub at [https://github.com/MaximeCarriere/cogninest](https:/github.com/MaximeCarriere/cogninest). This ensures full transparency and reproducibility, allowing other researchers to replicate, modify, and extend our work for further investigations in neurocomputational modelling. Simulations were executed on the HPC cluster of Freie Universität Berlin, using 6 CPU cores of Intel Xeon processors under AlmaLinux 8 with the Slurm batch system (Bennett et al. 2020).
