# Correction: A brain-constrained neural model of cognition and language withNEST: transitioning from the Felix framework

**DOI:** 10.1007/s11571-026-10524-1

**Published:** 2026-08-27

**Authors:** Maxime Carriere, Fynn Dobler, Hans Ekkehard Plesser, Agata Feledyn, Rosario Tomasello, Thomas Wennekers, Friedemann Pulvermüller

**Affiliations:** 1 Department of Philosophy and Humanities Brain Language Laboratory, WE4 Freie Universität Berlin, 14195 Berlin, Germany; 2 Cluster of Excellence’ Matters of Activity. Image Space Material’, Humboldt Universität zu Berlin, 10099 Berlin, Germany; 3 School of Mind and Brain, Humboldt Universität zu Berlin, 10117 Berlin, Germany; 4 Einstein Center for Neurosciences, 10117 Berlin, Germany; 5 School of Engineering, Computing and Mathematics, Faculty of Science and Engineering, University of Plymouth, PL4 8AA Plymouth, UK; 6 Department of Data Science, Faculty of Science and Technology, Norwegian University of Life Sciences, Ås, Norway; 7 Institute for Advanced Simulation (IAS-6), Research Centre Jülich, Jülich, Germany; 8 Käte Hamburger Kolleg: Cultures of Research (c:o/re), RWTH Aachen University, Aachen, Germany

**Correction to: Cognitive Neurodynamics (2026) 20:48**

10.1007/s11571-026-10415-5

In this article, Fig. 4 appeared as too small, the incorrect and correct version (Fig. 4) is as follows:

Incorrect version Fig. 4:

**Figure:**
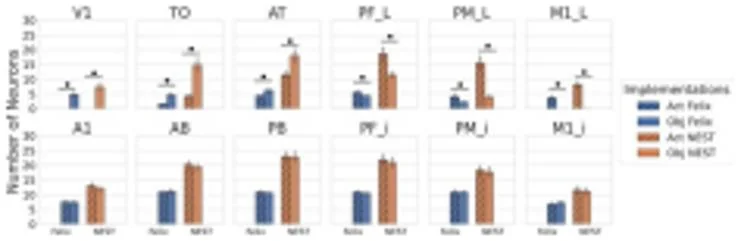

Correct version Fig. 4:

**Figure:**
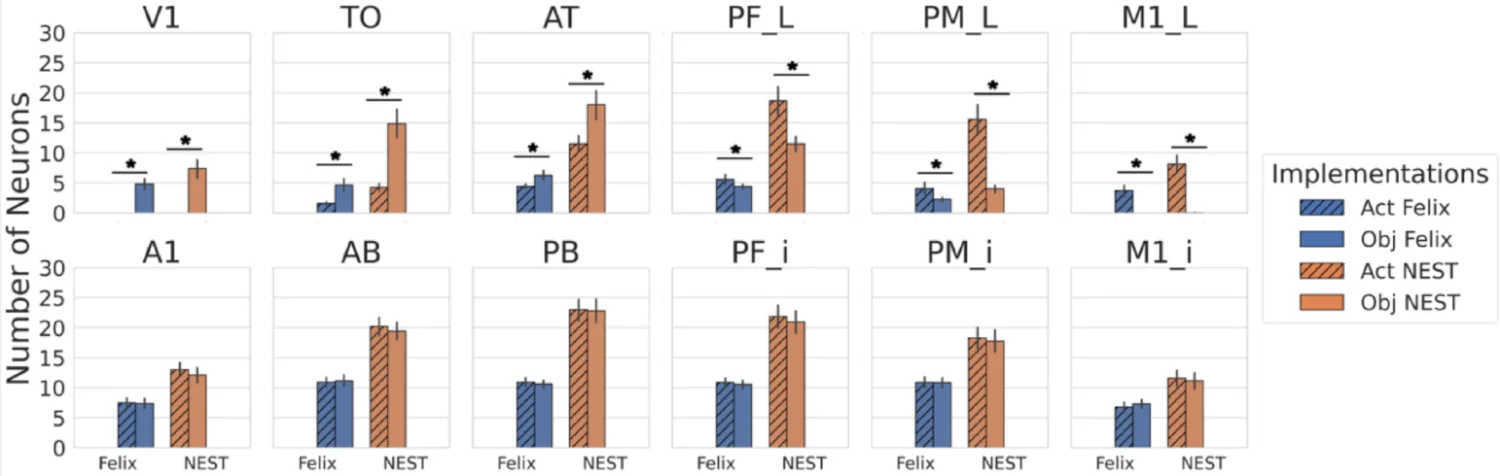

The original article has been corrected.

