# Supplementary material for: A brain-constrained neural model of cognition and language with NEST: transitioning from the Felix framework
Source: Cogn Neurodyn. 2026 Feb 6;20(1):48. doi: 10.1007/s11571-026-10415-5 (PMC12881243; doi:10.1007/s11571-026-10415-5)
Supplement: Supplementary file 1 — Supplementary Material 1 [file 11571_2026_10415_MOESM1_ESM.docx]

Supplementary Materials

Learning Rate Analysis (NEST)


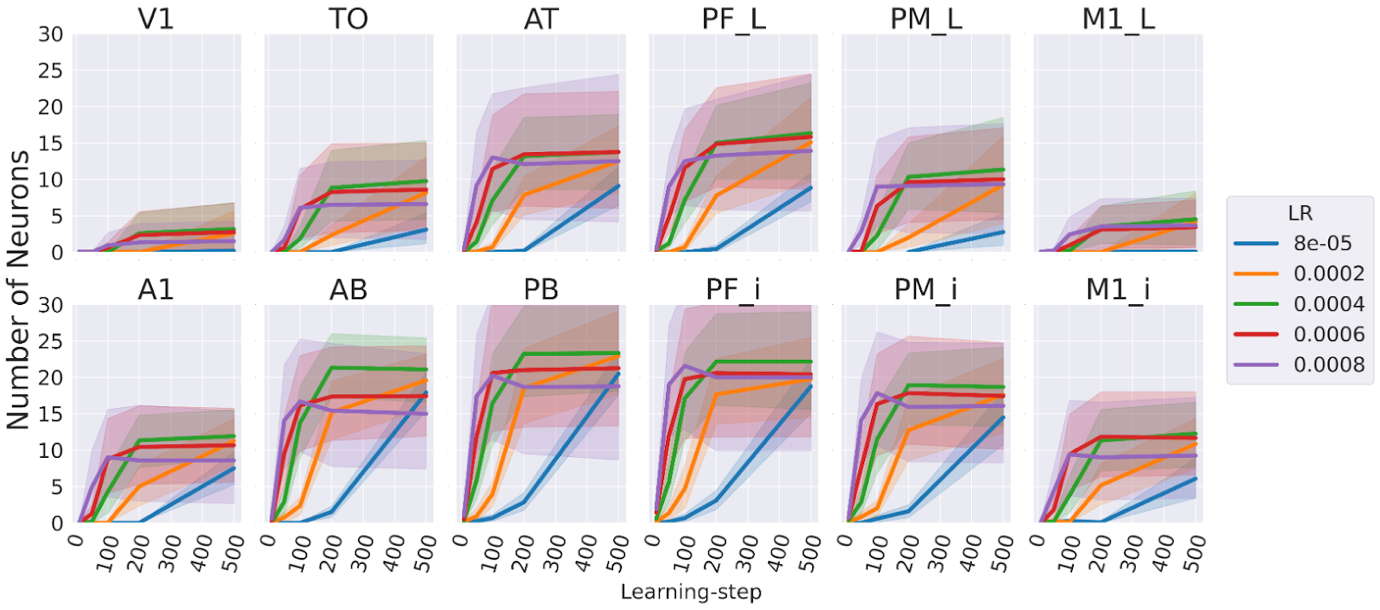


**Figure S1**: Cell Assembly Size over Learning Episode for different Learning Rate with the NEST implementation. Higher learning rates systematically increased the number of neurons per assembly in NEST, demonstrating that assembly size is strongly modulated by excitability-related parameters.

Theta - Analysis (NEST)


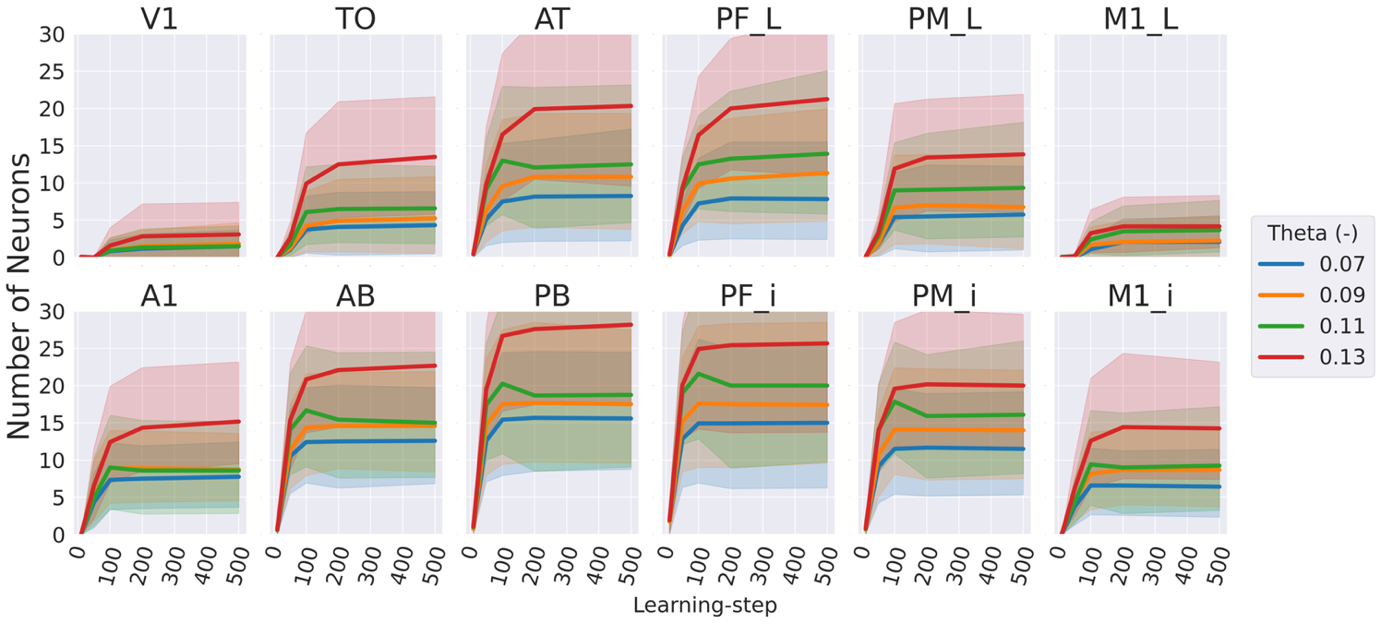


**Figure S2**: Cell Assembly Size over Learning Episode for different Theta (-)  with the NEST implementation. Higher thresholds led to larger assemblies, consistent with prolonged excitatory activity and delayed inhibitory onset in NEST. This further supports the interpretation that differences in assembly size arise from altered excitation–inhibition dynamics rather than learning performance per se.


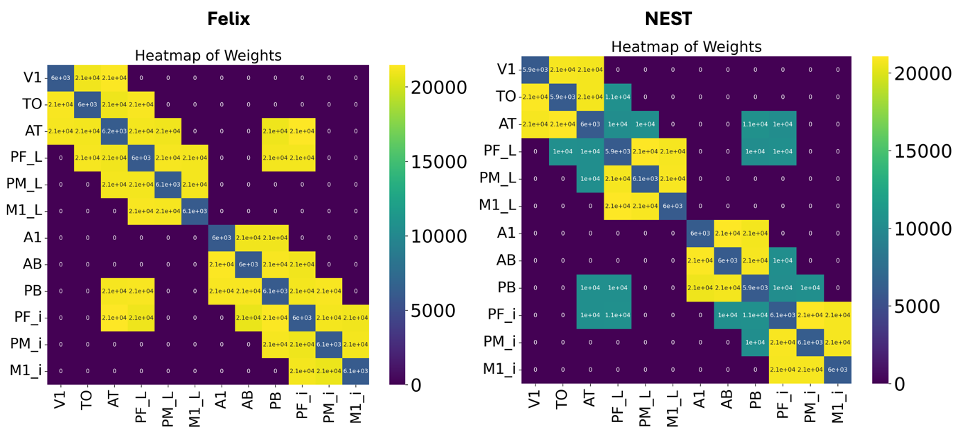


**Figure S3**: Matrix of Inter-area connection of Felix (left) vs NEST (right). The number of connections is also indicated in each square.

| Connectivity structure of the modeled cortical areas | |  |
| --- | --- | --- |
| Modelled Areas | References |  |
| **Between-Area Connectivity (black arrows)** | |  |
| **Peri-sylvian System** |  |  |
| **A1, AB, PB** | (Kaas & Hackett, 2000; Pandya, 1995; Rauschecker & Tian, 2000) |  |
| **PF_i_ , PM_i_ , M1_i_** | (Pandya & Yeterian, 1985; Young et al., 1994) |  |
| **Extra-sylvian System** |  |  |
| **V1, TO, AT** | (Bressler et al., 1993; Distler et al., 1993) |  |
| **PF_L_, PM_L_, M1_L_** | (Arikuni et al., 1988; Dum, 2005; Dum & Strick, 2002; Lu et al., 1994; Pandya & Yeterian, 1985; Rizzolatti & Luppino, 2001) |  |
| **Between System** |  |  |
| **AT, PB** | (Gierhan, 2013) |  |
| **PF_i,_ PF_L_** | (Yeterian et al., 2012) |  |
| **Long Distance Cortico-Cortical Connections (purple arrows)** | |  |
|  |  |  |
| **Peri-sylvian System** |  |  |
| PFi, PB | (Catani et al., 2005; Makris & Pandya, 2009; Meyer et al., 1999; G. J. M. Parker et al., 2005; Paus et al., 2001; J. K. Rilling et al., 2008; L. M. Romanski et al., 1999) |  |
| **Extra-sylvian System** | |  |
| AT, PFL | (Bauer & Jones, 1976; Chafee & Goldman-Rakic, 2000; Eacott & Gaffan, 1992; Fuster et al., 1985; A. Parker & Gaffan, 1998; Ungerleider et al., 1989; Webster et al., 1994) |  |
| **Between System** |  |  |
| PB, PFL | (Pandya & Barnes, 1987; L. m. Romanski et al., 1999; L. M. Romanski et al., 1999) |  |
| AT, PFi | (Pandya & Barnes, 1987; Petrides & Pandya, 2009; J. K. Rilling, 2014; L. M. Romanski, 2007; Ungerleider et al., 1989; Webster et al., 1994) |  |
| **High order “Jumping” Links (blue arrows)** | |  |
| Peri-sylvian System | (J. Rilling et al., 2012; J. K. Rilling et al., 2008; J. K. Rilling & van den Heuvel, 2018; Thiebaut de Schotten et al., 2012) |  |
|  | (Pandya & Yeterian, 1985; Young et al., 1994) |  |
|  |  |  |
| A1, PB |  |  |
| PB, PMi | (J. K. Rilling et al., 2008; Saur et al., 2008) |  |
| AB, PFi | (Deacon, 1992; Guye et al., 2003; Kaas & Hackett, 2000; Petrides & Pandya, 2009; Rauschecker & Scott, 2009; L. m. Romanski et al., 1999; Young et al., 1994) |  |
| PFi, M1i | (Deacon, 1992; Guye et al., 2003; Young et al., 1994) |  |
| Extra-sylvian System | (Thiebaut de Schotten et al., 2012) |  |
| V1, AT | (Catani, 2003; Wakana et al., 2004) |  |
| AT, PML | (Bauer & Fuster, 1978; Chafee & Goldman-Rakic, 2000; Fuster et al., 1985; Pandya & Barnes, 1987; Seltzer & Pandya, 1989) |  |
| TO, PFL | (Bauer & Jones, 1976; Fuster & Jervey, 1981; Makris & Pandya, 2009; Pandya & Barnes, 1987; Seltzer & Pandya, 1989) |  |
| PFL, M1L | (Deacon, 1992; Guye et al., 2003) |  |

Table **S1**: Area Connectivity and References


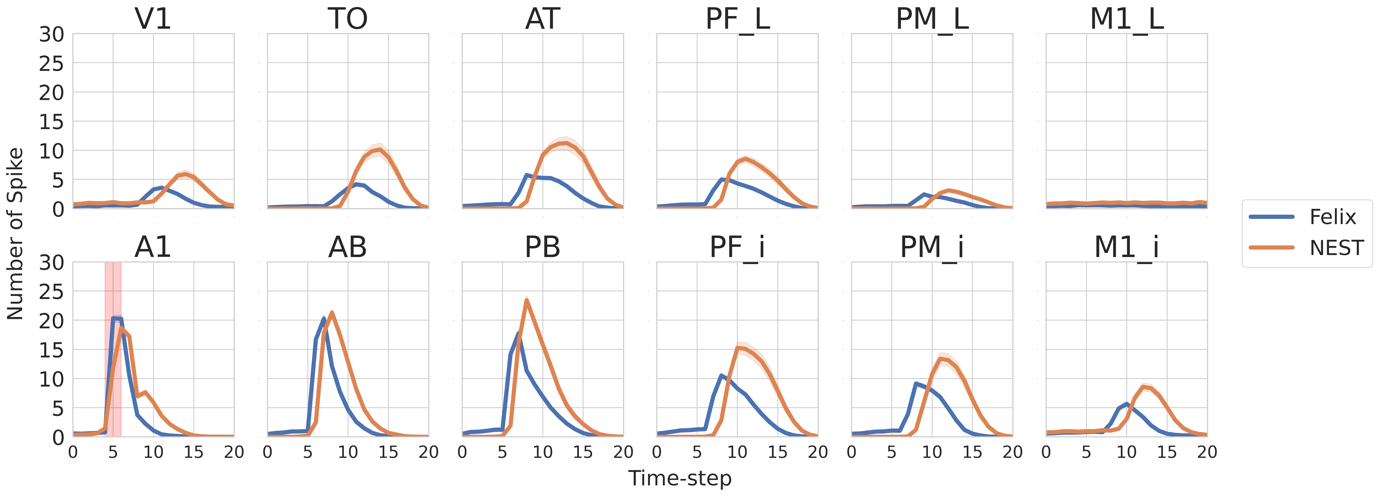


**Figure S4**: **Time course of activation following auditory (A1) stimulation for an object word across cortical areas in NEST and Felix implementations.** The number of spikes is plotted over simulation time steps for each cortical area, separately for NEST (orange) and Felix (blue). The stimulation period in A1 is indicated by the shaded red area. Activity spreads from A1 to perisylvian regions (AB, PB, PFi, PMi, M1i) and further into extra-sylvian lateral prefrontal and sensorimotor regions (V1, TO, AT) in both implementations.


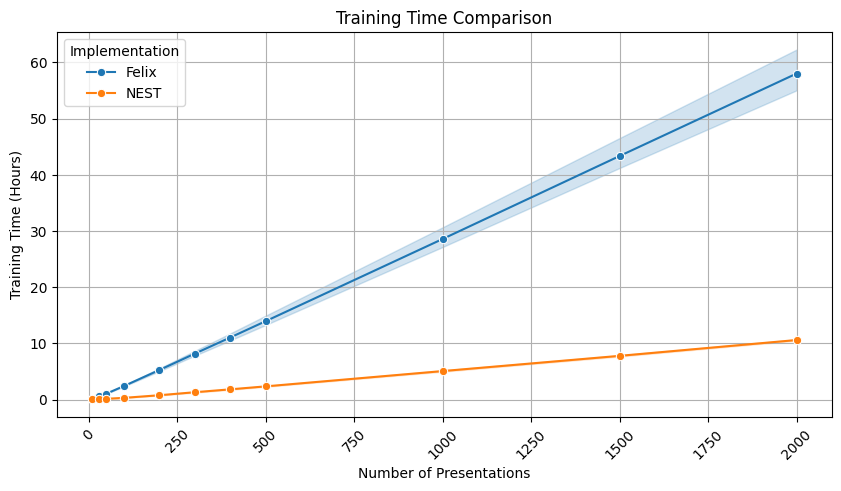
**Figure S5: Training time comparison between Felix and NEST implementations.** Mean training time (in hours) is plotted against the number of input pattern presentations for both implementations. NEST (orange) demonstrates a nearly sixfold reduction in runtime compared to Felix (blue), with linear scaling across training size. Shaded areas indicate standard error across runs. All simulations were executed on the high-performance computing (HPC) cluster of Freie Universität Berlin, using 6 CPU cores of Intel Xeon processors under AlmaLinux 8 with the Slurm batch system.
